# Interstitial microbial communities of coastal sediments are dominated by *Nanoarchaeota*

**DOI:** 10.3389/fmicb.2025.1532193

**Published:** 2025-02-18

**Authors:** Simone Brick, Jutta Niggemann, Anja Reckhardt, Martin Könneke, Bert Engelen

**Affiliations:** School of Mathematics and Science, Institute for Chemistry and Biology of the Marine Environment (ICBM), Carl von Ossietzky Universität Oldenburg, Oldenburg, Germany

**Keywords:** subterranean estuary (STE), high-energy beach, epipsammon, interstitial communities, DPANN superphylum, *Nanoarchaeota*, candidate phyla radiation

## Abstract

Microbial communities in subsurface coastal sediments are highly diverse and play an important role in nutrient cycling. While the major fraction of microorganisms in sandy sediments lives as epipsammon (attached to sand grains), only a small fraction thrives in the interstitial porewaters. So far, little is known about the composition of these free-living microbial communities. In the subsurface of the sandy beach, investigated in this study, we compared the archaeal and bacterial community structures within sediments and corresponding porewaters applying 16S rRNA gene sequencing. We found that the free-living prokaryotes only had a proportion of about 0.2–2.3% of the bulk communities, depending on the pore space. The interstitial microbial communities showed a small overlap with the attached fraction of 4–7% ASVs, and comprised a unique composition of 75–81% ASVs found exclusively in the porewaters. They were more diverse than the respective sediment-attached fraction and showed a much higher archaea-to-bacteria ratio. The archaea were mainly affiliated to *Nanoarchaeota* of the DPANN superphylum, with a relative abundance up to 50% of the interstitial communities. The bacterial fraction included several species related to the Candidate Phyla Radiation (CPR). Both prokaryotic lineages are known to have small cell sizes, comprising not-yet cultured species with unidentified metabolic functions. Our findings were supported by the investigation of an adjacent tidal flat, showing a similar trend. Thus, our results indicate the presence of distinct interstitial microbial communities in the subsurface of coastal sediments. This natural enrichment of not-yet cultured *Nanoarchaeota* and members of the CPR provides the opportunity for targeted metagenomic analyses or even isolating members of these groups for further metabolic characterization.

## Introduction

Coastal areas play an important role for global nutrient cycling and microorganisms are the main drivers of these biogeochemical processes (Moore, 2010; Beck et al., 2017; Degenhardt et al., 2020; Ruiz-González et al., 2022). Worldwide, about one third of ice-free coasts are classified as sandy beaches (Luijendijk et al., 2018). These are permeable systems that enable advective porewater flow which enhances the flux of dissolved constituents and their turnover by microorganisms. The continuously introduced flux of freshly produced organic matter and electron acceptors like oxygen by seawater is orders of magnitude higher than in diffusive systems (Holtappels et al., 2016). Mineralization of the organic matter results in an enrichment of nutrients in the porewaters (Reckhardt et al., 2015). However, many coastal areas are additionally impacted by terrestrial groundwater flow (Simmons, 1992; Robinson et al., 2007a; Moore, 2010), which is low in oxygen and transports aged organic matter, nitrogen, phosphorus and silicate into marine sediments (Waska et al., 2021; Burnett et al., 2003; Cho et al., 2018). Generally, the terrigenous organic matter exhibits low bioavailability, whereas organic matter in the seawater endmember is primarily produced by phytoplankton and appears to be more labile (Waska et al., 2021). In these coastal aquifers, seawater and terrestrial groundwater mix in a so-called subterranean estuary (STE) concomitant with chemical transformations before it discharges into the sea as submarine groundwater discharge (SGD) (Moore, 2010; Huettel et al., 1998). While these flow patterns are quite stable over time in low-energy systems, steadily changing boundary conditions influence the flow path of the porewater at high-energy beaches. Thus, tidal cycles, waves or changing beach morphology lead to a highly dynamic distribution of fresh and saline groundwater (Greskowiak and Massmann, 2021; Greskowiak et al., 2023).

So far, few studies have examined microbial communities in aquifers of sandy beaches (Archana et al., 2021). For instance, Hong et al. (2019) analyzed bulk sediment communities of the upper meter of a STE located at Gloucester Beach in Virginia, United States. The sediment communities had proportions of up to 25% archaea, which were mostly related to *Panarchaeota* and *Crenarchaeota*. In general, the microbial abundance is depending on the surface area that is available for colonization and the composition as well as the exposure to circulating porewater (Ahmerkamp et al., 2020). An analysis of microbial communities attached to single sand grains showed, that they were highly diverse, but shared a core community that covered more than 50% of OTUs from the bulk sediments (Probandt et al., 2017). The diversity was conditioned by micro niches within the sediment, caused by the shapes of the sand grains and porewater flux. The flow path followed a hydraulic gradient, that was influenced by flow- and topography-induced pressure gradients, tidal cycles, waves and sediment compaction (Santos et al., 2012).

**Figure 1:**
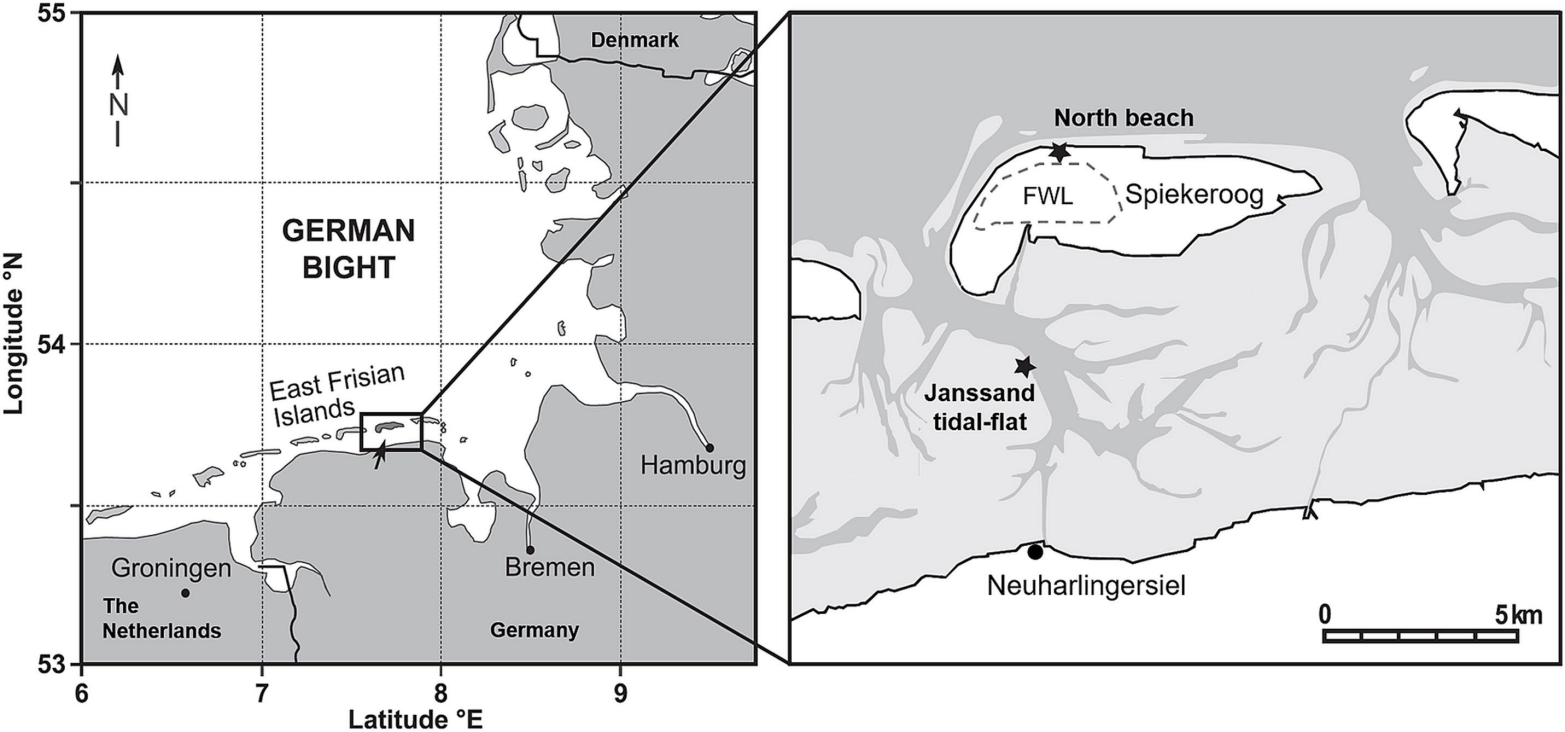
Sampling sites at the north beach of the German barrier island Spiekeroog and the Janssand tidal-flat (modified after Reckhardt et al., 2015). Based on the tidal range of 2.7 m and mean significant wave heights of 1.4 m, the north beach of Spiekeroog is classified as a high-energy beach. The light gray area on the right map represents the tidal-flats which fall dry at low-tide. The dotted line indicates the location of the freshwater lens (FWL). On both sites, sediments and corresponding porewaters were collected from the surface, 0.1, 0.3, 0.5 and 1 meter below ground surface (mbgs). Additionally, deep porewater and sediment samples were recovered from 6, 12, 18 and 24 mbgs at Spiekeroog beach.

Previous investigations on our study site, the high-energy beach of the German Island Spiekeroog (Figure 1), showed that the core community composition of the sediment-attached fraction of the upper meter consisted of generalists which did not change significantly by season and depth (Degenhardt et al., 2020). However, different areas of Spiekeroog beach still showed a number of site-specific organisms, exhibiting highest variations at the high-water line. Accordingly, the prokaryotic communities at sites affected by SGD showed a higher evenness than the communities in other areas of the beach (Degenhardt et al., 2021). A site-specific microbial community composition was also found within STEs from two other high-energy beaches located in Baiona Bay in Spain, when exclusively comparing porewaters using 16S rRNA analyses (Calvo-Martin et al., 2022). Those communities were composed of highly abundant cosmopolitans and locally limited rare taxa. The main environmental factors that impacted the microbial community compositions were oxygen and dissolved organic matter. Overall, interstitial porewaters might show a high spatial heterogeneity concerning biomass, activity and diversity of microbial communities, which was for example shown in a study by Ruiz-González et al. (2022) who examined a Mediterranean STE.

All aforementioned studies lack a direct comparison of sediment-attached (epipsammon) and interstitial microbial communities, which is a general knowledge gap for coastal aquifers, as emphasized in a review article by Ruiz-González et al. (2021). While the majority of microorganisms in these aquifers are living as epipsammon (Rusch et al., 2001), interstitial bacteria and archaea comprise only a very small fraction of the entire community and have often been overlooked in the past. A recent study by Moncada et al. (2024) analyzed sandy seafloor sediments and differentiated between porewater associated, loosely attached and firmly attached microorganisms. They found dissimilar bacterial communities in the different fractions with the majority of the cells being firmly attached to the sediment. Due to the different metabolic activities of the three fractions, the authors suggest that they occupy distinct niches within the surface sediment. In freshwater aquifers, several studies found high percentages of ultra-small bacteria and archaea such as *Nanoarchaeota* and members of the Candidate Phyla Radiation (CPR or *Patescibacteria*) (He et al., 2021; Castelle et al., 2018; Anantharaman et al., 2016; Chaudhari et al., 2021; Luef et al., 2015). Small cell sizes can be an advantage in oligotrophic environments, as the surface-to-volume ratio is higher and can increase the efficiency of nutrient uptake. However, many ultra-small microorganisms have small genomes that lack genes for essential metabolic pathways (Ghuneim et al., 2018). Thus, they often live as symbionts with other bacteria or archaea, which was also reported for several members of the CPR and the DPANN superphylum, an acronym of the names of the initially assigned phyla (*Ca. Diapherotrites*, *Ca. Parvarchaeota*, *Ca. Aenigmarchaeota*, *Nanoarchaeota* and *Ca. Nanohaloarchaeota*) (Huber et al., 2002; Huber et al., 2003; Castelle et al., 2015; Nelson and Stegen, 2015; Castelle et al., 2018; Chiriac et al., 2022; Rinke et al., 2013). Bacteria and archaea of both radiations are distributed worldwide and may comprise approximately half of the overall microbial diversity (Castelle et al., 2018). Yet, the majority of members of these phyla are so far uncultured and their role in the environment remains unclear.

In our study, we separately analyzed the epipsammon and the interstitial microbial community compositions within the STE of the northern beach of Spiekeroog. This beach has high-energy conditions with semi-diurnal tides with a range of 2.7 m and mean significant wave heights of 1.4 m (Greskowiak and Massmann, 2021). In the subsurface of the island, a freshwater lens has formed which is recharged by infiltrating precipitation (Röper et al., 2012). In the beach area, groundwater from the lens discharges and mixes with recirculating seawater in the STE (Moore, 1999; Greskowiak et al., 2023). Due to tide and wave driven water fluxes in the STE, an upper saline plume with fast circulating seawater forms in the intertidal zone. This hydraulic feature is accompanied by a surficial mixing zone, a saltwater wedge and a freshwater discharge tube near the low-water line (Robinson et al., 2007b). The dynamic biogeochemistry with varying salt concentrations, nutrient availability and organic matter compositions makes this aquifer a specific habitat for microorganisms (Calvo-Martin et al., 2022). Several studies have previously been conducted at this site, but the interstitial microbial communities have not been analyzed so far. Furthermore, little is known about prokaryotes that thrive in deeper parts of the STE. Thus, a STE observatory was established on Spiekeroog beach in the frame of the interdisciplinary research unit “Dynamic deep subsurface of high-energy beaches” (DynaDeep) (Massmann et al., 2023). The observatory includes three multilevel groundwater wells at the dune base (ML1), close to the high-waterline (ML2) and the low-water line (ML3). In the course of constructing these wells, sediment cores from the dune base and the high-water line were recovered and analyzed to explore the composition of the epipsammon and the interstitial communities. We first examined a set of sediment and porewater samples from the upper meter of Spiekeroog beach and compared it to another set of samples from the beach subsurface at depths of 6, 12, 18 and 24 meters below ground surface (mbgs). Our hypotheses were (i) that the porewater community comprises a fraction of the sediment-attached community, e.g., by washing off loosely attached microorganisms and (ii) that the porewaters harbor specific interstitial microbial communities, independent from the epipsammon. As a further comparison, we took samples from Janssand (Figure 1), a sand flat in the back barrier area south of Spiekeroog Island (Beck et al., 2009). In the tidal flat, the microbial activity is higher compared to the beach due to enhanced primary production in the water column and high sedimentation rates. The tidal-flat sediments are only influenced by advective flow of marine porewaters and not by terrestrial groundwater (Reckhardt et al., 2015). In this study, the prokaryotic compositions of samples from Spiekeroog beach and site Janssand were analyzed applying Illumina 16S rRNA gene sequencing to identify common features of both, the epipsammon and interstitial fractions.

## Methods

### Sampling sites: Spiekeroog beach and Janssand tidal-flat

Spiekeroog is one of the east Frisian barrier islands off the North-west German coastline in the Wadden Sea (Figure 1). The island extends about 10 km west–east and 2 km north–south with the northern beach facing the open sea. The morphology of the intertidal zone of Spiekeroog beach is characterized by a ridge and runnel system parallel to the shoreline, which is dynamic in space and time (Anthony et al., 2005; Waska et al., 2019). Freshwater exfiltrates in the runnel and near the low water line (Waska et al., 2019; Grünenbaum et al., 2020). For our study, porewater and sediment samples of the upper meter (in the following referred to as shallow samples) were collected from the runnel in June 2021. The sediment was completely water-saturated during all tidal cycles. Sediment and porewater from deeper layers of the beach were sampled in the following year close to the high-water line about 60 m apart from the shallow sampling site. Another set of samples was collected on Janssand tidal-flat, which is part of the backbarrier tidal-flat area of Spiekeroog. While this sandbank is covered with up to two meters of water during high tide, it is exposed to air for about 6 h during low tide (Beck et al., 2009). The samples were taken during low tide at the slope towards the tidal channel at the eastern tidal-flat margin, which was intensively studied biogeochemically over the past two decades (Seidel et al., 2012; Zielinski et al., 2022; Beck et al., 2009). The upper meter of this tidal flat is dominated by fine sand and is only influenced by seawater infiltration and advective porewater flow, but not by fresh groundwater flow from the hinterland (Reckhardt et al., 2015).

### Recovery of sediments and porewaters and sample preparation

Three sediment cores were taken from the upper meter of the runnel on Spiekeroog beach in June 2021 as replicates. The cores, that had a diameter of 8 cm and a length of 1.50 m, were drilled at a distance of approximately 40 cm from each other in aluminum tubes using a jackhammer. One additional 1.5 m long sediment core was recovered in October 2022 from Janssand tidal-flat using a vibrocorer. To obtain undisturbed surface samples, 50 cm cores were taken manually. In the laboratory, the cores were cut across for sampling at 0.1, 0.3, 0.5 and 1 m. Deep subsurface sediments from Spiekeroog beach were sampled in the course of installing the groundwater well ML2 for the DynaDeep project (Massmann et al., 2023). One continuous 24 m long core was taken by dry drilling according to DIN EN ISO 22475-1 close to the high-water line. The core was obtained in sections of 1 m length in PVC liners which had a diameter of 10 cm. In the laboratory, the liners of all core sections were cut crosswise at a length of 50 cm using a tube-cutter. To prevent contamination, the cores were broken up without using a cutting device to obtain undisturbed sediment surfaces. Sediment samples were taken from the most pristine center of the whole-round cores (Lever et al., 2006). In our study, four depths were analyzed to which we refer as 6, 12, 18 and 24 meters below ground surface (mbgs). From the respective depths of all cores, 5 mL sediment were sampled in sterile cut-off syringes in 3 replicates and stored for DNA-extraction at −20°C. For total cell counts, 0.5 mL of the sediment were transferred to a 2 mL reaction tube, mixed with 1 mL 3% glutardialdehyde (GDA) and incubated for 30 min at 4°C. To wash off the GDA, the samples were centrifuged for 5 min at 13000 rpm, the supernatant was removed and 1 mL Tris-acetate-EDTA (TAE) buffer was added to the sediment pellet. This step was repeated twice, except that the last washing step was conducted using a TAE-ethanol mix (1:1) to avoid the formation of ice crystals during storage at −20°C.

Porewater of the shallow beach and the tidal flat was collected from the corresponding depths using stainless steel push-point samplers (outer diameter 5 mm, inner diameter 3 mm) that were connected to gas washing bottles with a teflon tube and subsequently refilled to high-density polyethylene bottles. For measurements of nutrients and dissolved organic carbon (DOC) from the porewater of the shallow Spiekeroog samples, porewater was collected with polyethylene syringes directly from the push-point sampler. Samples for salinity measurements were measured using a Multi 3,630 IDS with a TetraCon 925 conductivity probe (WTW, Munich, Germany). No chemical analyses were done on the Janssand tidal-flat samples. Porewaters from the deeper layers of the high-water line were sampled from the multilevel-well ML2, using a submersible pump (“Gigant,” Eijkelkamp, Giesbeek, Netherlands). The groundwater well was screened in depth of 5–6 mbgs, 11–12 mbgs, 17–18 mbgs and 23–24 mbgs with slots that had a size of 0.3 mm to filter out sediment particles (Massmann et al., 2023), thus covering the corresponding depths to the sediment. As for the sediment, we refer to the samples as 6, 12, 18 and 24 mbgs. Sampling took place in December 2022, as this was the earliest time point providing stable conditions for microbiological analyses after setting up and equilibration of the wells. Prior to sampling, the double well volume was pumped out and discarded to obtain pristine porewaters. Subsequently the oxygen saturation and salinity were measured using the Multi 3,630 IDS with a TetraCon 925 conductivity probe and a FDO 925 O_2_ probe (WTW, Munich, Germany). The probes were placed in a flow through cell, that was directly attached to the tubing of the pump.

In the laboratory, 9.6 mL water was amended with 400 μL GDA (25%) in triplicates, incubated for 30 min at 4°C and subsequently frozen at −20°C for cell counting. For nutrient measurements, 1.5 mL sample were mixed with 4.5 μL HgCl_2_. 10 μL distilled HNO_3_ were added to 1 mL of the samples for manganese and iron measurements. For DOC and total dissolved nitrogen (TDN) measurements, 15 mL sample were filtered through pre-combusted glass microfibers (GMF, 2 μm, Whatman, Little Chalfont, United Kingdom) and glass fiber filters (GF/F, 0.7 μm, Whatman, Little Chalfont, United Kingdom) into HDPE bottles and immediately acidified with HCL to pH 2. 250 mL of porewater was sequentially filtered with 3 μm and 0.1 μm pore size polycarbonate filters (Ø 47 mm, Whatman, Little Chalfont, United Kingdom). The 0.1 μm filters were kept for DNA-extraction. Concentrations of nitrate, nitrite, ammonium, phosphate, iron and manganese were determined as described in Reckhardt et al. (2024). DOC and TDN were analyzed using high-temperature catalytic oxidation (Shimadzu TOC-VCPH, Duisburg, Germany).

### Total cell counts

For total cell counts of the sediment community, cells were detached by incubating 0.5 cm^3^ of sediment for 15 min in 990 μL phosphate buffered saline (PBS buffer, pH 7.4) (Sambrook, 1989) and 10 μL of 0.001 M pyrophosphate. Subsequently, samples were sonicated two times for 3 min with cooling in between for 1 min. The supernatant was transferred to a sterile reaction tube and the remaining sediment pellet was homogenised with 1 mL of PBS buffer by shaking. The supernatant was combined with the first one. This step was repeated five times (Lunau et al., 2005). Depending on cell density, aliquots of the supernatant were diluted 1:10, 1:20 or 1:50 with PBS buffer and filtered on black polycarbonate-filters (Ø 25 mm, pore size 0.2 μm, Whatman, Little Chalfont, United Kingdom), which were then transferred to microscope slides. For counting porewater samples, 5 mL porewater/GDA mix were directly filtered without pre-treatment. The cells on the filters were stained using 10 < μl SybrGreen-mix (5 μL SybrGreen, 5 μL ascorbic acid (1 M), 200 μL Moviol). For each sample, a minimum of 300 cells (10–20 fields of view) were counted using epifluorescence microscopy (Leitz DM R, Leica, Wetzlar, Germany).

### Determination of porosity and density

Porosity and density of the sediment samples were determined using 10 g sediment of the respective depths. The sediment was weighed into pre-weighed glass cylinders and filled up to 14 mL with distilled water. After weighing again, the volume of sediment was calculated by subtracting the volume of the water from the total volume, assuming a density of the water of 1 g/cm^3^. Density was calculated using the following formula:


$$\rho =\frac{m_{\mathrm{sediment}}}{V_{\mathrm{sediment}}}\;\left[\frac{g}{cm^{3}}\right]$$

For calculation of the porosity, the wet sediment was transferred to pre-weighed glass petri dishes, making sure to transfer the whole sediment. After complete drying at 80°C, the sediment was re-weighed, and the porosity was calculated as follows:


$$\Phi =\frac{V_{\mathrm{porewater}}}{V_{\mathrm{sediment}}}\;\left[\frac{cm^{3}}{cm^{3}}\right];\mathrm{with}\;0\langle \Phi \rangle 1$$

### DNA-extraction, amplification of 16S rRNA genes, and sequencing

DNA was extracted with phenol-chloroform after a modified protocol of Lueders et al. (2004) and Gabor et al. (2003) using 0.5 g of each sediment sample, or a half of the 0.1 μm filters containing the interstitial communities, respectively. As a blank, 250 mL ultrapure water was filtered and the filter was processed corresponding to the samples. Samples were vortexed in 750 μL PTN buffer and incubated for 15 min with 200 μg lysozyme and 150 μg proteinase K at 37°C. After addition of 100 μL sodium dodecyl sulfate (SDS, 20%), samples were incubated at 65°C, while shaking at 500 rpm. Subsequently, 100 μL phenol-chloroform-isoamylalcohol (25:24:1) were added followed by bead beating for 45 s and spinning down at 7500 rpm for 5 min. The supernatant was transferred to a sterile 2 mL vial. The remaining sediment or filter was again vortexed with 150 μL PTN buffer, and after bead beating (20 s) and centrifuging (5 min at 7500 rpm) the supernatant was pooled with the previously obtained supernatant and extracted with the equal volume of phenol-chloroform-isoamylalcohol. After manual shaking, the samples were centrifuged 4 min at 14000 rpm and the aqueous phase was extracted with the equal volume of chloroform-isoamylalcohol (24:1). This mixture was spun down for 4 min at 14000 rpm and the upper phase was mixed with the double volume of polyethylene glycol (PEG). Samples were incubated over night before the DNA was spun down at 14000 rpm for 30 min. The PEG was decanted and the DNA-pellet was washed with ice-cold ethanol (70%). After centrifuging for 5 min at 14000 rpm, the ethanol was discarded and the DNA was dried at room temperature. Finally, the DNA was dissolved in 30 μL PCR-grade water. To amplify the 16S rRNA gene, primers for sequencing the V4 and V5 region were used according to forward 515F-Y (5′-GTG YCA GCM GCC GCG GTA A-3′); reverse 926R (5’-CCG YCA ATT YMT TTR AGT TT-3′) (Parada et al., 2016). This primer set has a similar coverage across bacteria (84.5%) and archaea (81.0%). The sequencing reaction was initiated with 2 μL of 1:10 diluted target DNA or 2 μL PCR-grade water as a negative control, mixed with 5 μL Phusion-HF-buffer (5x), 2 μL dNTPs (2.5 mM), 0.75 μL of each primer (10 μM), 0.5 μL MgCl2 (50 mM), 0.25 μL Phusion-taq polymerase (2 U/μl) and 13.75 μL PCR-water. Amplification was performed on a Mastercycler nexus X2 (Eppendorf, Wesseling-Berzdorf, Germany) including the following steps: activation at 98°C for 3 min, 25 cycles comprising denaturation at 98°C for 45 s, annealing at 50°C for 45 s and elongation at 72°C for 90 s. The final elongation step was performed at 72°C for 5 min. Magnetic beads were used to purify the 16S amplicons following a protocol by Illumina (2013). Subsequently, an Index-PCR was performed using Illumina barcodes “IDT-Illmn DNA–RNA UD Indexes Tagmentation.” For this PCR, 20 μL PCR-grade water were mixed with 10 μL Phusion-HF-buffer, 0.5 μL MgCl2 (50 mM), 4 μL dNTPs (2.5 mM), 0.5 μL Phusion-taq polymerase (2 U/μl), 10 μL of the Indices and 5 μL DNA-template. The PCR was performed on a Mastercycler nexus X2 (Eppendorf, Wesseling-Berzdorf, Germany) using the following program: Activation at 95°C for 3 min, 8 cycles comprising denaturation at 95°C, annealing at 55°C and elongation at 72°C for 30 s each. The final elongation step was performed at 72°C for 5 min. Subsequently, a second clean-up step with magnetic beads followed (Illumina, 2013). Sequencing was performed with the Illumina NextSeq 1,000 sequencer (Illumina, Berlin, Germany).

### Bioinformatics and statistical analyses

The generated 16S rRNA data were processed using DADA2 (Callahan et al., 2016) according to Tebbe et al. (2022), cutting the forward and the reverse sequence at 240 bp and 200 bp, respectively. For taxonomic classification, the database SILVA138 was used. Sequences that were related to mitochondria and chloroplasts were omitted in the subsequent analyses. Datasets of the respective layers from triplicate cores from Spiekeroog beach were pooled to equalize individual variations. The sequences were deposited at the European Nucleotide Archive under the accession number PRJEB82696. For further analyses, read abundances were converted to relative abundances. All statistical analyses were performed in R v. 2022.07.0 (RStudio-Team, 2022). Shannon diversity, richness and evenness were calculated based on ASVs using the “vegan” package. Non-metric multidimensional scaling (NMDS) (k = 3, 999 permutations) was used to visualize Bray-Curtis distances of the microbial abundances in the sediment and porewater samples of the respective sites. The analysis of similarity (ANOSIM) with 999 permutations was performed to assess statistical significance regarding the dissimilarity of these samples. Venn diagrams were created using the R package “venn.” To display taxonomic variations and to identify indicator species of the investigated communities, the analysis was performed using sequencing results based on the highest obtainable taxonomic level. Indicator species were identified using multi-level pattern analysis within the R package “indicspecies” and visualized using “reshape2.”

## Results

### Porewater chemistry and total cell counts

Overall, the investigated sites showed varying chemical compositions and sediment features (Supplementary Figure S1; Supplementary Table S1). While the porosity of the shallow Spiekeroog beach sediments from the runnel shifted between 11 and 39% in the upper meter, it decreased from 24% at 6 mbgs to 18% at 24 mbgs along the depth profile of the deep Spiekeroog samples collected close to the high-water line. At the Janssand tidal-flat, porosity was in the range of 30 to 36%, showing highest porosity of 53% in the more muddy sediment layer at 1 mbgs. DOC concentrations were almost twice as high in the shallow beach samples as in the deep porewater and highest at the beach surface (160 μM). Unfortunately, no detailed chemical data for the Janssand samples were recorded in the current study, but are available from previous investigations (Seidel et al., 2014; Reckhardt et al., 2015; Beck et al., 2008). Published DOC concentrations were about ten times higher compared to the beach samples. Salinity was around 31 in the shallow Spiekeroog beach samples and decreased from 29 to 18 in the deep aquifer from 6 to 24 mbgs. At Janssand salinity was at seawater concentration and oxygen was depleted at 0.1 mbgs and below. While no oxygen data are available for the shallow Spiekeroog beach samples the presence of dissolved Fe^2+^ (89 μM in 1 mbgs) and Mn^2+^ (1.9–4.2 μM) indicate anoxic conditions in the runnel at 0.1 mbgs and below. The depth profile of the deep Spiekeroog samples collected close to the high-water line showed oxic conditions in 6 mbgs (saturation of 76%) and beginning NO_3_ and Mn-reducing conditions at 12 mbgs. This is in accordance to the concentration of dissolved iron which was <1 μM at 6 and 12 mbgs, and further increased at in deeper layers (16–17 μM). Manganese concentrations were also higher at 18 mbgs and 24 mbgs than above (0.5 μM).

Cell counting showed that the interstitial communities comprised only a very small fraction of the bulk community (Tab. S2). In the upper meter of Spiekeroog beach, cell numbers were in the range of 10^7^ cells · cm^−3^ bulk sediment (added epipsammon and interstitial communities). Taking the porosity into account, the interstitial communities exhibited cell numbers in the range of 10^5^ cells · cm^−3^ bulk sediment, only. In the deep Spiekeroog samples, about 10^6^ cells · cm^−3^ bulk sediment were counted with 10^3^–10^5^ cells deriving from interstitial microorganisms. The epipsammon of the upper meter of the Janssand tidal flat had considerably higher cell numbers that were in the magnitude of 10^8^–10^9^ cells · cm^−3^ bulk sediment.

### Shallow Spiekeroog beach samples and tidal-flat communities are dominated by similar phyla

**Figure 2:**
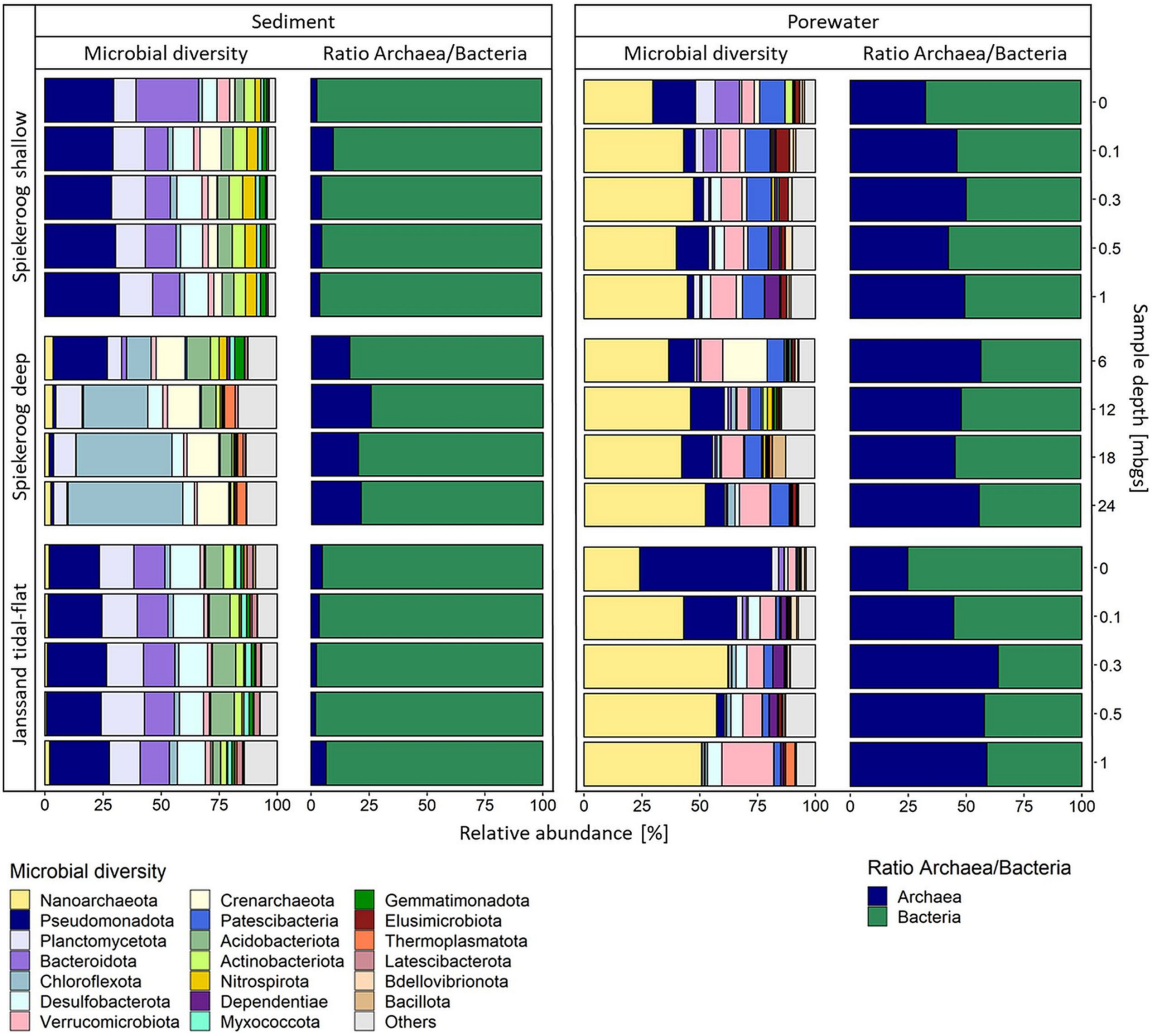
Microbial community composition of the shallow and deep samples from Spiekeroog beach and Janssand tidal flat. The colors represent different prokaryotic phyla in the respective samples. Each bar shows the community composition of one sample depth. The barplots show the 20 most abundant phyla over all samples. All further phyla are summed up as “Others.” The green-blue barplots show the ratio of archaea and bacteria (blue = archaea, green = bacteria).

Sequencing of all investigated samples resulted in 3,758,627 16S rRNA gene sequences with an average of 134,242 reads per sample after quality control, comprising a diversity of 49,691 amplicon sequence variants (ASVs) assigned to 81 phyla and candidate divisions. The analysis of the 16S rRNA genes of all shallow samples showed significant differences in the archaea-to-bacteria ratios between the sediment and porewater communities (Figure 2). While the sediment-attached archaea in the shallow samples of Spiekeroog beach had proportions of 2.5–9.5% of the whole community and 2.0–6.5% at Janssand tidal-flat, their relative abundance was up to 50% in the Spiekeroog porewaters and even 64% at a depth of 0.3 mbgs and in deeper layers of Janssand tidal-flat. In the data sets of both sites, the proportion of archaea was lowest in the surface samples, but still had proportions of 33% at Spiekeroog beach and 25% at Janssand tidal-flat, respectively.

An analysis of the 16S rRNA genes on the highest available taxonomic level revealed that the epipsammon community structure generally appeared to be quite uniform (Supplementary Figures S4A–C), while the interstitial communities showed more distinct compositions. The archaeal fraction of the sediment-attached community from Spiekeroog beach mainly consisted of *Crenarchaeota*, while the interstitial community was dominated by *Nanoarchaeota* of the order *Woesearchaeales* with percentages of 30–47% of the entire microbial community (Figure 2). Furthermore, the interstitial communities had a proportion of about 10% of bacteria affiliated to the CPR, namely *Patescibacteria*, which showed a minor relative abundance in the Janssand tidal-flat samples (up to 4% rel. abund.). The most abundant bacterial phyla of the epipsammon from both sites were *Bacteroidota*, *Planctomycetota Desulfobacterota* and *Pseudomonadota*. The latter and the *Verrucomicrobiota* were also most prevalent constituents of the bacterial porewater communities from Spiekeroog beach. *Bacteroidota* were abundant taxa in the surface layer (10.5% rel. abund.) and at a depth of 0.1 mbgs (6% rel. abund.), but had percentages of less than 0.5% in deeper porewater samples, only. At Janssand tidal-flat, *Verrucomicrobiota* were also present in all porewater samples with the highest relative abundance at 1 mbgs (22% rel. abund.). Here, besides the high proportion of *Nanoarchaeota*, the porewater communities were dominated by *Pseudomonadota* in the surface layer and at 0.1 mbgs.

Overall, the microbial communities from the shallow porewaters of Spiekeroog beach exhibited a higher Shannon diversity and species richness (H´ = 6.01–7.23, R = 753–3,202) compared to the sediment communities (H´ = 5.47–6.36, R = 417–1,079) of the respective depths (Supplementary Figure S2). In contrast, samples from Janssand tidal-flat showed the opposite trend (sediment: H´ = 7.63–7.77, R = 8,722–10,340; porewater: H´ = 4.59–6.47, R = 694–1814). Except for the 1 meter sample from Spiekeroog beach, the evenness was slightly higher in the sediment samples (E = 0.90–0.91) than in the porewaters (E = 0.87–0.91). This was also true for the Janssand tidal-flat communities from the upper 10 cm and at 1 m depth. However, in all other samples from Janssand, the porewater communities exhibited a higher evenness of E = 0.70–0.90 than the sediment communities (E = 0.82–0.84).

### Communities from deep beach samples are dominated by *Chloroflexota* and *Nanoarchaeota*

In the epipsammon of the deep Spiekeroog samples, the archaea-to-bacteria ratios were 17–26%, which is much higher than for the shallow samples (2.5–9.5%) (Figure 2). While most archaea were related to the *Crenarchaeota*, *Thermoplasmatota* were additionally present in the samples below 6 mbgs. In the porewater communities, archaea had relative abundances of up to 57%, mainly affiliated to *Nanoarchaeota*. The most prevalent bacterial phyla in the porewaters were *Pseudomonadota*, *Verrucomicrobiota* and *Patescibacteria*, which corresponded to the shallow porewater community. The sediment-attached community in the deep samples differed significantly from those of the shallow sediment layers. Here, the community showed an increasingly high proportion of *Chloroflexota* (10% at 6 mbgs to 50% at 24 mbgs). Still, the community composition from 6 mbgs was different from the deeper samples, which was probably caused by changing redox conditions below 6 mbgs (Supplementary Figure S1). Like in the shallow samples, the Shannon diversity and species richness were higher in the porewater communities (H´ = 6.70–7.42, R = 2046–4,920) than in the sediments (H´ = 4.84–6.49, R = 179–1,156). The evenness of the sediment communities was highly similar over all depths of the deep Spiekeroog samples (E = 0.88–0.94) and the porewater communities had a slightly lower evenness (E = 0.80–0.93) (Supplementary Figure S2).

### Site specific community compositions on higher taxonomic level

**Figure 3:**
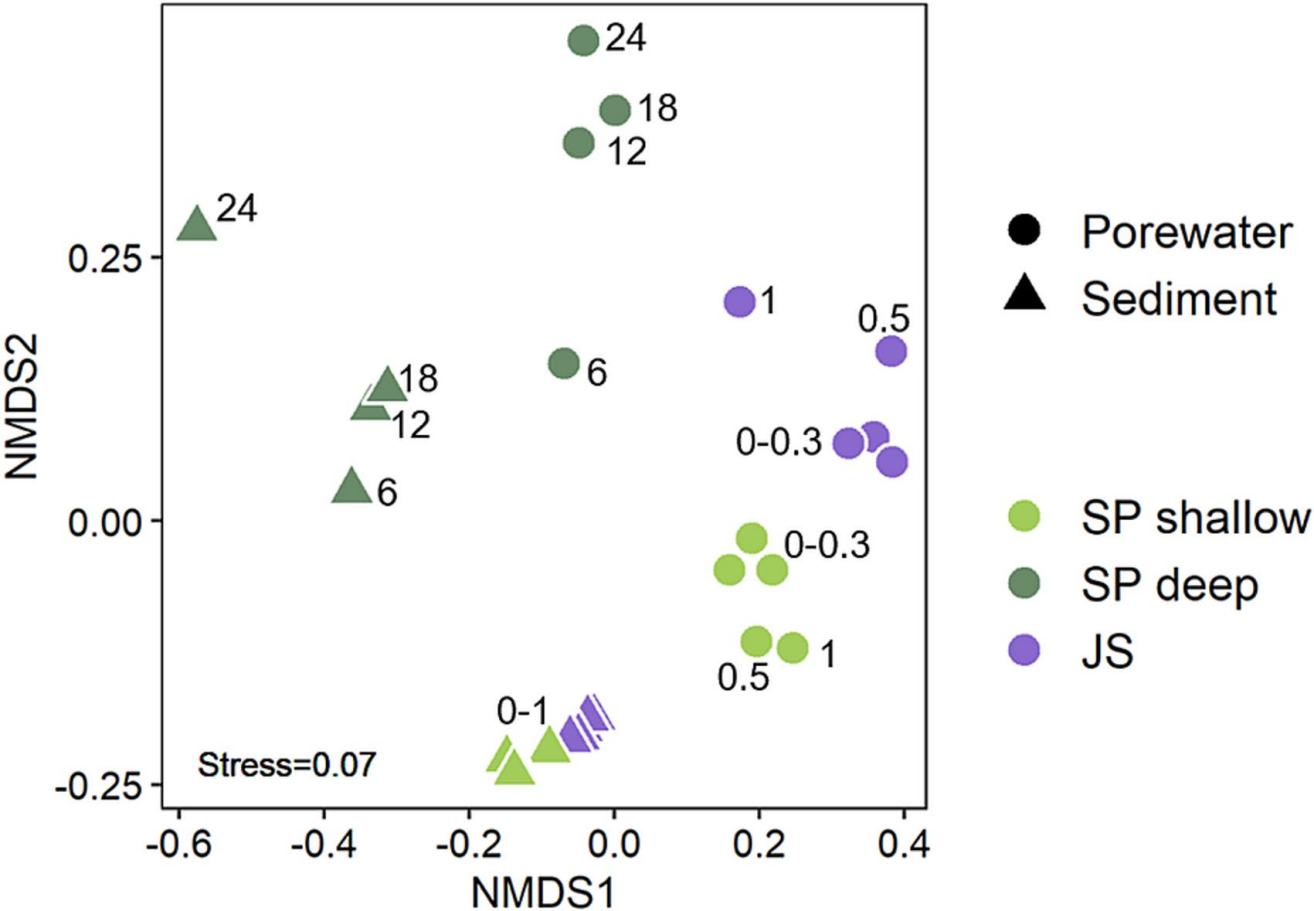
Non-metric multidimensional scaling (NMDS) of porewater and sediment communities of Spiekeroog beach and Janssand tidal flat. The numbers in the plot display the sample depth in meter below ground surface (mbgs). The analysis of similarity (ANOSIM) showed significant differences between the sites (*p* = 0.001), attached and free-living communities (*p* = 0.001) and depths (*p* = 0.004).

**Figure 4:**
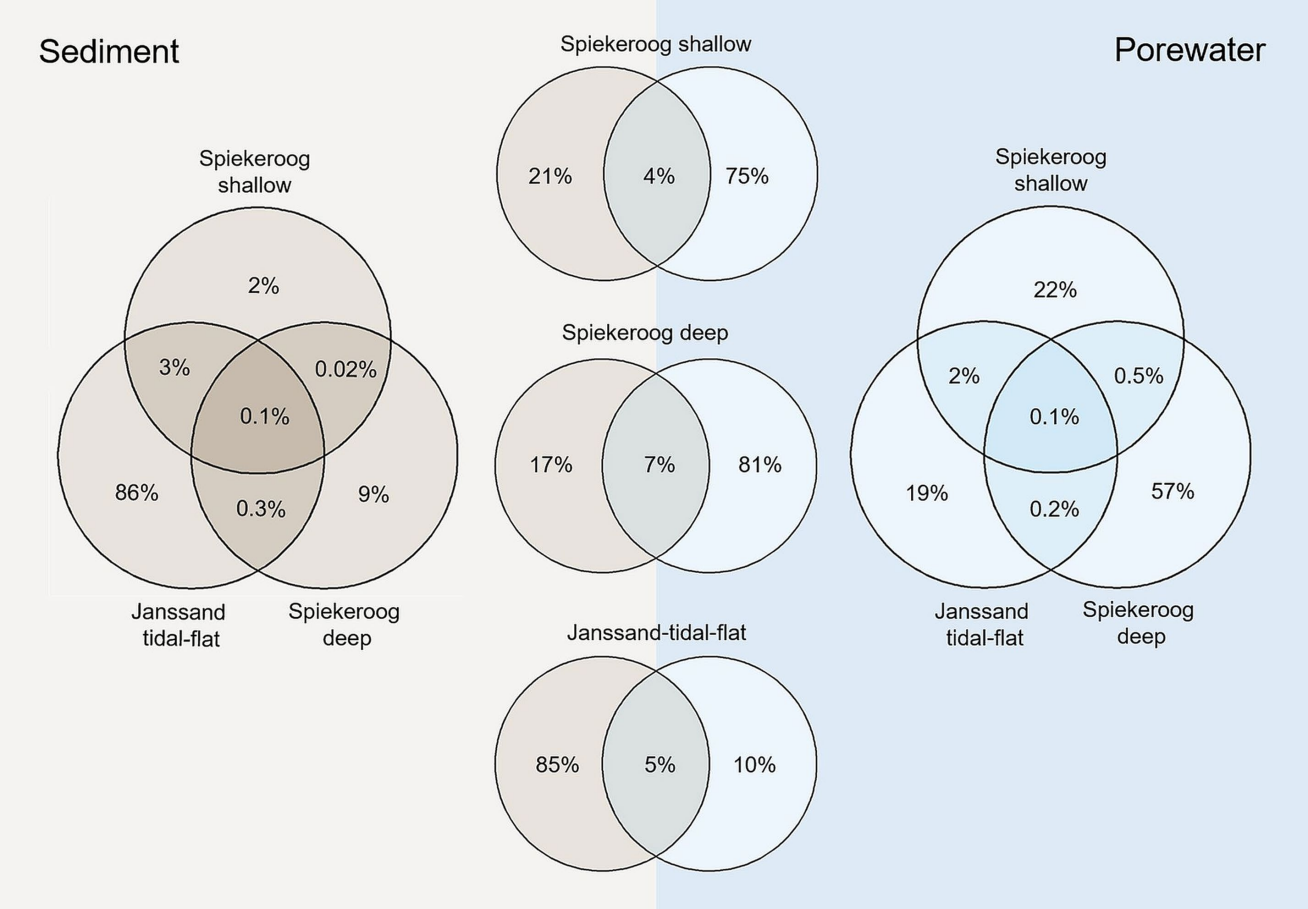
Venn diagrams showing the percentage of shared ASVs in the samples of the shallow and deep beach communities and the tidal-flat community of the epipsammon (beige) and the interstitial porewaters (blue). The three Venn diagrams in the center show the percentage of ASVs that were detected in the porewater or sediment on the respective site.

Non-metric multidimensional scaling (NMDS) revealed significant differences between the interstitial and epipsammon communities (*p* = 0.001) (Figure 3). Comparing the number of ASVs of the interstitial communities and the epipsammon for the Spiekeroog beach samples and Janssand tidal-flat separately, only 4–7% were shared between both sample types (Figure 4). While the majority of individual ASVs was very high in the porewater of Spiekeroog beach (75% in shallow and 81% in deep samples), the Janssand epipsammon comprised 85% ASVs, that were not detected in the porewaters of the tidal-flat. Although the interstitial communities were mostly dominated by the same phyla, the overall community structures were significantly different between the sites (*p* = 0.001). The same was true for all sediment samples. Here, the shallow samples from Spiekeroog beach and Janssand tidal-flat formed separate clusters with low internal variabilities. The sediment communities of the individual depths of the deep Spiekeroog samples, in turn, exhibit a much higher internal variability. The porewater communities showed a similar pattern, but in both shallow Spiekeroog and Janssand samples the communities from 0.5–1 mbgs differed slightly from the layers above (0–0.3 mbgs). These differences between the communities of individual depths were also significant (*p* = 0.004). While the sediment communities of all samples comprised 30,831 different ASVs, 21,394 ASVs were identified in all porewater samples. Based on the number of sediment-derived ASVs, 86% were exclusively found in the Janssand tidal-flat and 0.1% formed the core community of all sediment samples. The porewater communities of both sites shared 0.1% ASVs, while the deep Spiekeroog beach comprised 57% of all ASVs, that were exclusively found in these layers (Figure 4). An indicator species analysis confirmed a strong association of *Nanoarchaeota* and members of the *Patescibacteria* to the porewater communities. This analysis showed, that 65 taxa were strongly associated to the shallow Spiekeroog porewater with significant *p*-values (<0.05) (Supplementary Figure S3). Among those were two members of the *Nanoarchaeota*, which had high indicator values (> 0.99) and a high abundance in the porewater as well as 28 bacteria related to the *Patescibacteria*, and Lineage IV of the *Elusimicrobiota*. Those bacteria and archaea are characterized by very small cell sizes. In the communities of the deep Spiekeroog samples, two *Nanoarchaeota* were among the highly associated indicator species and 24 members of the *Patescibacteria*.

## Discussion

We found specific interstitial communities that only shared a minor fraction with the corresponding epipsammon. They comprised a unique composition of ASVs related to ultra-small bacteria and archaea, which were detected exclusively in the porewaters. The majority of these microorganisms is so far uncultured and their role in the environment remains to be identified.

### Composition of the epipsammon is impacted by changing redox conditions with depth

The sediment-attached communities thriving in shallow and deep sediment layers of Spiekeroog beach exhibit distinct community structures. The epipsammon of the upper meter harbors a variety of bacteria related to *Pseudomonadota, Planctomycetota* and *Bacteroidota*, which comprised major fractions of the investigated communities. This corresponds to our previous findings on the upper meter of Spiekeroog beach where we analyzed bulk sediments at different seasons (Degenhardt et al., 2020). This was initially not expected, as the beach morphology was highly dynamic over the course of several years, characterized by dramatic reworking of the sediments and even changing the beach elevation in decimeter to meter ranges. Material from the sublittoral is regularly deposited in the intertidal zone or is washed off by waves and tides at this high-energy beach. The presence of the observed core community of generalists can be interpreted as an adaptation to this ever-changing environmental conditions and the steady alteration of the surface layer. However, the epipsammon of the deeper beach layers is not that exposed to beach-morphology changes, but to changing redox conditions and dynamics in the STE. This is indicated by differences in the community composition of the epipsammon between 6 mbgs and the deeper layers where oxygen was already depleted. The deep sediments of Spiekeroog beach are clearly dominated by members of the *Chloroflexota*. These bacteria are often found in the deep subsurface in high abundances and are thus characteristic for this habitat (Wilms et al., 2006a,b; Inagaki et al., 2006; Hug et al., 2013). Even though this bacterial lineage also comprises many not-yet cultured organisms which could not be physiologically characterized, there is limited information available to infer their metabolic niche space. Members of the *Chloroflexota* are found in anoxic environments where they can ferment sugars and are involved, e.g., in acetate formation. Furthermore, genes for nitrate reduction were found in several members of this phylogenetic group (Hug et al., 2013). Their occurrence was previously observed in environments that have a poor availability and quality of organic matter (Wilms et al., 2006a). This fits to the nutritional conditions in the deep STE of Spiekeroog beach. Here, organic matter is already reworked so that recalcitrant DOM is prevalent as bioavailable carbon source (Abarike et al., 2024).

Compared to the northern beach of Spiekeroog, Janssand tidal-flat is a much more active system. This is reflected by the cell numbers, that were 2–3 orders of magnitude higher than in the samples from Spiekeroog beach. The sequence of redox processes (Froelich et al., 1979) is highly condensed along the depth profile of the tidal flat in comparison to low-active sites (Engelen and Cypionka, 2009). The redox sequence at Spiekeroog beach, in turn, is spread to a greater depth, but is regularly changing due to dynamic flow patterns in the STE (Greskowiak et al., 2023). While the oxygen availability generally depends on the hydrodynamics of the system, at Janssand tidal-flat, it penetrates not more than a few millimeters to centimeters into the sediment due to a high microbial activity (Jansen et al., 2009). At our sampling site, the eastern margin of the tidal flat, microbial communities are fueled by high amounts of DOC. Here, DOC concentrations are about ten times higher compared to the beach samples due to high sedimentation rates (Reckhardt et al., 2015; Beck et al., 2009). Despite the differences in the porewater chemistry, the core community of the epipsammon of the tidal flat is quite similar to the shallow beach samples (Figure 3). Similar to the Janssand tidal flat margin, the beach runnel represents an anoxic discharge zone. Compared to the anoxic zones below 6 meter in the seawater infiltration zone, the shallow runnel sediments likely contain more degradable/labile organic matter, e.g., due to regular sediment relocation (Reckhardt et al., 2024). Also, the diversity of the epipsammon was much higher at the Janssand margin than in the beach samples, as reflected in an extremely high number of rare taxa, that solely occurred in the epipsammon of the tidal flat (Figure 4). This high diversity is likely to be supported by the availability of high concentrations of DOC in the tidal-flat margin. While in our study we only analyzed samples from the upper meter, previous studies on Janssand tidal-flat found a shift from high abundant *Pseudomonadota* in the upper sediment layers to *Chloroflexota* dominated communities below 2 m depth (Wilms et al., 2006a,b). Combined with our findings on the shallow and deep Spiekeroog beach samples we conclude that the epipsammon of the beach has a similar core community compared to that of the tidal flat.

### Interstitial communities are impacted more by discharging porewater than infiltrating seawater

Microorganisms in the interstitial waters represent only a tiny fraction of the bulk community. However, these porewater communities do not consist of detached sediment bacteria and archaea but are significantly different from the epipsammon. Our analyses showed that only a small fraction of ASVs are shared between sediment and porewater communities of the different locations (Figure 4). In the Spiekeroog beach samples, the majority of individual ASVs were abundant in the porewater. Interstitial communities of all depth of Spiekeroog beach were dominated by ultra-small and many so-far uncultured microorganisms. While up to 10 % were related to *Patescibacteria*, up to half of the entire community was related to *Nanoarchaeota* of the DPANN superphylum. *Nanoarchaeota* were previously detected in high abundances in freshwater aquifers (Groult et al., 2023; Gios et al., 2023), but according to our findings they are also thriving in porewaters with high salinity. Interestingly, the porewater communities differed clearly from the seawater, even at the surface layers of both sites. Seawater from the shoreline of Spiekeroog beach was sampled repeatedly over the course of one year and samples were collected and analyzed corresponding to the porewater as described above (data not shown). The most distinct difference was the lack of *Nanoarchaeota* and *Patescibacteria* in the seawater. This is surprising as both sampling sites were strongly impacted by seawater infiltration. While the shallow Spiekeroog beach sediments were collected from the runnel, which had a direct connection to the sea and quickly fills up with seawater with upcoming high-tide, the tidal flat is covered with seawater in every tidal cycle. Thus, the pore space of both sites is frequently seawater-saturated, but also impacted by discharging porewater from the STE of Spiekeroog beach or at the tidal-flat margins during low tide, respectively. According to our results, the interstitial communities of both sites are seemingly more impacted by discharging porewater than by infiltrating seawater, even at the surface. This corresponds to our chemical analyses of the porewater. While the high-water line is a seawater infiltration zone, where oxygen-saturated water infiltrates into greater depth, the runnel is a discharge area with Mn/Fe-reducing water. The generally high percentage of ultra-small microorganisms in our porewater samples corresponds to results of He et al. (2021). In their study the authors performed genome-resolved metagenomic analysis of microbial communities from one agriculturally impacted and seven pristine groundwater samples. Even though the proportion of DPANN archaea was about ten times lower than in our samples, the authors found high proportions, but also site-specific diversities of DPANN archaea and *Patescibacteria*. We also calculated a strong association of both phylogenetic groups in the indicator species analysis of all porewater samples (Supplementary Figure S3) and still detected significant differences (Figure 3), e.g., by the comparably lower relative abundances of *Patescibacteria* within the tidal-flat communities.

### The role of interstitial *Nanoarchaeota* and members of the CPR in sandy sediments

All *Nanoarchaeota* in our samples were related to the order *Woesearchaeales* independently from sampling site and depth. Our sampling site on Spiekeroog beach provides oligotrophic conditions, especially in the deeper subsurface. Here, compounds introduced with the seawater are depleted and the labile organic matter is already degraded, so that recalcitrant organic matter remains. Additionally, the material of the upper layers is undergoing steady deposition and erosion. Thus, the sampling site does not provide stable conditions over time but is variable in nutrient and salt concentrations. *Nanoarchaeota* are presumably well adapted to these conditions so that they can outcompete other microorganisms in this habitat. According to Castelle et al. (2018), who analyzed about 1,000 genomes reconstructed from several metagenomics-based studies, the majority of prokaryotes related to the DPANN and CPR are anaerobes. This fits partly to our findings as the limitation of electron acceptors in the deep beach samples favors microorganisms that can thrive by fermentation. Moreover, we also found *Nanoarchaeota* in high percentages in the porewaters of Janssand tidal flat, where the nutrient availability is considerably higher. In the tidal flat, fermentation represents an important part of anaerobic degradation processes and provides substrates for, e.g., sulfate reducers and methanogens (Graue et al., 2012). According to Castelle et al. (2018), CPR and DPANN organisms may play important roles in carbon cycling, as they are presumably able to degrade complex carbon compounds, e.g., from plant material. Furthermore, they are predicted to produce lactate, formate and ethanol via fermentation, which can then be utilized by other microorganisms.

The occurrence of both phylogenetic groups even in the oxic part of the porewaters from Spiekeroog beach point towards alternative lifestyles. Many members of these two groups are predicted to depend on other bacteria or archaea as they have minimal metabolic pathways, such as the lack to synthesize some fatty acids, amino acids and nucleotides. Thus, it is assumed that they scavenge these compounds from other organisms or dead cell material (Castelle et al., 2018). The only isolated member of the *Nanoarchaeota* is *Nanoarchaeum equitans*, which is suggested to be an obligate symbiont that grows attached to the archaeon *Ignicoccus hospitalis* (Huber et al., 2002). *Nanoarchaeota* might be able to attach to different hosts, as it was also proposed in a previous study for the majority of the *Patescibacteria* by Chaudhari et al. (2021). According to that study, this lifestyle enables them to adapt to their respective environmental conditions. The authors conclude, that the symbiotic lifestyle of both groups may be well suitable to stable, oligotrophic environments, but that a parasitic lifestyle cannot be ruled out. Limited pore space is favoring the attachment to host cells or the uptake of dead cell material. Thus, their relevant characteristics contributing to the ecosystem may include the mineralization of dead microbial biomass and recalcitrant organic matter (Castelle et al., 2018), regardless of the redox conditions and related porewater chemistry.

### Joint features of the epipsammon and interstitial communities

We aimed to compare the microbial communities of sediment-attached and corresponding free-living fractions and to find overlaps in their community structures. Instead, our study revealed significant differences in the compositions of these communities, most prominently by the high archaea-to-bacteria ratio in the porewaters, representing a natural enrichment of archaea. While most of the microorganisms live attached to the sediment grains, this part of the bulk community consists of a highly adapted core community, depending on the supply of electron donors and acceptors from the interstitial porewaters. The interstitial communities move with the porewater and always face the same water body, while the epipsammon thrive on particles and are exposed to changing conditions due to different composition of the groundwater that flows by. The communities of the interstitial waters were dominated by *Nanoarchaeota* at all depth at both sites, but also revealed distinct differences in their composition (Figure 3). Due to these striking differences in the community compositions of the interstitial porewaters and the epipsammon, we have to reject our initial idea to use the porewater community as a proxy for the bulk community. Furthermore, the high percentage of ultra-small microorganisms in the porewaters that are presumably exhibiting a symbiotic or parasitic lifestyle with so far unknown hosts makes it difficult to evaluate their role in the respective ecosystem. As we found the *Nanoarchaeota* in all porewater samples independently from sampling site and depth, they seem to be highly adaptive to their respective environment or they might not rely on one specific host. Also, metagenomic approaches aiming to shed light on their roles in the ecosystems still reveal many knowledge gaps. Castelle et al. (2018) stated, that approximately half of the genes in the genomes of CPR and DPANN organisms have yet unknown functions. These genes may be involved in interactions between host and symbiont (Castelle et al., 2018). Nevertheless, the natural enrichment of *Nanoarchaeota* in the porewater of Spiekeroog beach and the adjacent tidal flat provides the opportunity for targeted metagenomic analyses or even isolating them for further metabolic characterization.

## Data Availability

The datasets presented in this study can be found in online repositories. The names of the repository/repositories and accession number(s) can be found in the article/Supplementary material.

## References

[ref1] Abarike G. A. Brick S. Engelen B. Niggemann J. (2024). Different dissolved organic matter sources sustain microbial life in a Sandy Beach subterranean estuary – an incubation study. Front. Mar. Sci. 11:1501781. doi: 10.3389/fmars.2024.1501781

[ref2] Ahmerkamp S. Marchant H. K. Peng C. Probandt D. Littmann S. Kuypers M. M. M.. (2020). The effect of sediment grain properties and Porewater flow on microbial abundance and respiration in permeable sediments. Sci. Rep. 10:3573. doi: 10.1038/s41598-020-60557-7, PMID: 32107429 PMC7046789

[ref3] Anantharaman K. Brown C. T. Hug L. A. Sharon I. Castelle C. J. Probst A. J.. (2016). Thousands of microbial genomes shed light on interconnected biogeochemical processes in an aquifer system. Nat. Commun. 7:13219. doi: 10.1038/ncomms13219, PMID: 27774985 PMC5079060

[ref4] Anthony E. J. Levoy F. Monfort D. Degryse-Kulkarni C. (2005). Short-term intertidal Bar mobility on a ridge-and-Runnel Beach, Merlimont, northern France. Earth Surf. Process. Landf. 30, 81–93. doi: 10.1002/esp.1129

[ref5] Archana A. Francis C. A. Boehm A. B. (2021). The beach aquifer microbiome: research gaps and data needs. Front. Environ. Sci. 9:653568. doi: 10.3389/fenvs.2021.653568

[ref6] Beck M. Dellwig O. Liebezeit G. Schnetger B. Brumsack H.-J. (2008). Spatial and seasonal variations of Sulphate, dissolved organic carbon, and nutrients in deep pore waters of intertidal flat sediments. Estuar. Coast. Shelf Sci. 79, 307–316. doi: 10.1016/j.ecss.2008.04.007

[ref7] Beck M. Köster J. Engelen B. Holstein J. M. Gittel A. Könneke M.. (2009). Deep pore water profiles reflect enhanced microbial activity towards tidal flat margins. Ocean Dyn. 59, 371–383. doi: 10.1007/s10236-008-0176-z

[ref8] Beck M. Reckhardt A. Amelsberg J. Bartholomä A. Brumsack H.-J. Cypionka H.. (2017). The drivers of biogeochemistry in beach ecosystems: a cross-shore transect from the dunes to the low-water line. Mar. Chem. 190, 35–50. doi: 10.1016/j.marchem.2017.01.001

[ref9] Burnett W. C. Bokuniewicz H. Huettel M. Moore W. S. Taniguchi M. (2003). Groundwater and pore water inputs to the coastal zone. Biogeochemistry 66, 3–33. doi: 10.1023/B:BIOG.0000006066.21240.53

[ref10] Callahan B. J. Mcmurdie P. J. Rosen M. J. Han A. W. Johnson A. J. A. Holmes S. P. (2016). Dada2: high-resolution sample inference from Illumina amplicon data. Nat. Methods 13, 581–583. doi: 10.1038/nmeth.3869, PMID: 27214047 PMC4927377

[ref11] Calvo-Martin E. Teira E. Álvarez-Salgado X. A. Rocha C. Jiang S. Justel-Díez M.. (2022). On the hidden diversity and niche specialization of the microbial realm of subterranean estuaries. Environ. Microbiol. 24, 5859–5881. doi: 10.1111/1462-2920.16160, PMID: 36054689 PMC10087554

[ref12] Castelle C. J. Brown C. T. Anantharaman K. Probst A. J. Huang R. H. Banfield J. F. (2018). Biosynthetic capacity, metabolic variety and unusual biology in the Cpr and Dpann radiations. Nat. Rev. Microbiol. 16, 629–645. doi: 10.1038/s41579-018-0076-2, PMID: 30181663

[ref13] Castelle C. J. Wrighton K. C. Thomas B. C. Hug L. A. Brown C. T. Wilkins M. J.. (2015). Genomic expansion of domain Archaea highlights roles for organisms from new Phyla in anaerobic carbon cycling. Curr. Biol. 25, 690–701. doi: 10.1016/j.cub.2015.01.014, PMID: 25702576

[ref14] Chaudhari N. M. Overholt W. A. Figueroa-Gonzalez P. A. Taubert M. Bornemann T. L. V. Probst A. J.. (2021). The economical lifestyle of Cpr Bacteria in groundwater allows little preference for environmental drivers. Environ. Microbiome 16:24. doi: 10.1186/s40793-021-00395-w, PMID: 34906246 PMC8672522

[ref15] Chiriac M. C. Bulzu P. A. Andrei A. S. Okazaki Y. Nakano S. I. Haber M.. (2022). Ecogenomics sheds light on diverse lifestyle strategies in freshwater Cpr. Microbiome 10:84. doi: 10.1186/s40168-022-01274-3, PMID: 35659305 PMC9166423

[ref16] Cho H.-M. Kim G. Kwon E. Y. Moosdorf N. Garcia-Orellana J. Santos I. R. (2018). Radium tracing nutrient inputs through submarine groundwater discharge in the Global Ocean. Sci. Rep. 8:2439. doi: 10.1038/s41598-018-20806-2, PMID: 29403050 PMC5799265

[ref17] Degenhardt J. Dlugosch L. Ahrens J. Beck M. Waska H. Engelen B. (2020). Seasonal dynamics of microbial diversity at a Sandy high Energy Beach reveal a resilient Core Community. Front. Mar. Sci. 7:573570. doi: 10.3389/fmars.2020.573570

[ref18] Degenhardt J. Khodami S. Milke F. Waska H. Engelen B. Martinez Arbizu P. (2021). The three domains of life within the discharge area of a shallow subterranean estuary at a high Energy Beach. Front. Environ. Sci. 9:642098. doi: 10.3389/fenvs.2021.642098

[ref19] Engelen B. Cypionka H. (2009). The subsurface of tidal-flat sediments as a model for the deep biosphere. Ocean Dyn. 59, 385–391. doi: 10.1007/s10236-008-0166-1

[ref20] Froelich P. N. Klinkhammer G. P. Bender M. L. Luedtke N. A. Heath G. R. Cullen D.. (1979). Early oxidation of organic matter in pelagic sediments of the eastern equatorial Atlantic: Suboxic diagenesis. Geochim. Cosmochim. Acta 43, 1075–1090. doi: 10.1016/0016-7037(79)90095-4

[ref9001] Gabor E. M. De Vries E. J. Janssen D. B. (2003). Efficient recovery of environmental DNA for expression coning by indirect extraction methods. Fems Microbiology Ecology, 44, 153–163. doi: 10.1016/S0168-6496(02)00462-2 19719633

[ref21] Ghuneim L.-A. J. Jones D. L. Golyshin P. N. Golyshina O. V. (2018). Nano-sized and filterable Bacteria and Archaea: biodiversity and function. Front. Microbiol. 9:1971. doi: 10.3389/fmicb.2018.01971, PMID: 30186275 PMC6110929

[ref22] Gios E. Mosley O. E. Weaver L. Close M. Daughney C. Handley K. M. (2023). Ultra-small Bacteria and Archaea exhibit genetic flexibility towards groundwater oxygen content, and adaptations for attached or planktonic lifestyles. ISME Commun. 3:13. doi: 10.1038/s43705-023-00223-x, PMID: 36808147 PMC9938205

[ref23] Graue J. Kleindienst S. Lueders T. Cypionka H. Engelen B. (2012). Identifying fermenting Bacteria in anoxic tidal-flat sediments by a combination of microcalorimetry and ribosome-based stable-isotope probing. FEMS Microbiol. Ecol. 81, 78–87. doi: 10.1111/j.1574-6941.2011.01282.x, PMID: 22188432

[ref24] Greskowiak J. Massmann G. (2021). The impact of Morphodynamics and storm floods on pore water flow and transport in the subterranean estuary. Hydrol. Process. 35. doi: 10.1002/hyp.14050

[ref25] Greskowiak J. Seibert S. L. Post V. E. A. Massmann G. (2023). Redox-zoning in high-energy subterranean estuaries as a function of storm floods, temperatures, seasonal groundwater recharge and Morphodynamics. Estuar. Coast. Shelf Sci. 290:108418. doi: 10.1016/j.ecss.2023.108418

[ref26] Groult B. St-Jean V. Lazar C. S. (2023). Linking groundwater to surface discharge ecosystems: archaeal, bacterial, and eukaryotic community diversity and structure in Quebec (Canada). Microorganisms 11:1674. doi: 10.3390/microorganisms11071674, PMID: 37512847 PMC10384904

[ref27] Grünenbaum N. Ahrens J. Beck M. Gilfedder B. S. Greskowiak J. Kossack M.. (2020). A multi-method approach for quantification of in- and exfiltration rates from the subterranean estuary of a high Energy Beach. Front. Earth Sci. 8:571310. doi: 10.3389/feart.2020.571310

[ref28] He C. Keren R. Whittaker M. L. Farag I. F. Doudna J. A. Cate J. H. D.. (2021). Genome-resolved metagenomics reveals site-specific diversity of Episymbiotic Cpr Bacteria and Dpann Archaea in groundwater ecosystems. Nat. Microbiol. 6, 354–365. doi: 10.1038/s41564-020-00840-5, PMID: 33495623 PMC7906910

[ref29] Holtappels M. Neumann A. Ahmerkamp S. Marchant H. Winter C. (2016). Scaling of benthic fluxes in permeable sediments. Altern. Formats 45:47.

[ref30] Hong Y. Wu J. Wilson S. Song B. (2019). Vertical stratification of sediment microbial communities along geochemical gradients of a subterranean estuary located at the Gloucester Beach of Virginia, United States. Front. Microbiol. 9:3343. doi: 10.3389/fmicb.2018.03343 30687299 PMC6336712

[ref31] Huber H. Hohn M. J. Rachel R. Fuchs T. Wimmer V. C. Stetter K. O. (2002). A new phylum of Archaea represented by a Nanosized Hyperthermophilic symbiont. Nature 417, 63–67. doi: 10.1038/417063a, PMID: 11986665

[ref32] Huber H. Hohn M. J. Stetter K. O. Rachel R. (2003). The phylum Nanoarchaeota: present knowledge and future perspectives of a unique form of life. Res. Microbiol. 154, 165–171. doi: 10.1016/S0923-2508(03)00035-4, PMID: 12706504

[ref33] Huettel M. Ziebis W. Forster S. Luther G. W. (1998). Advective transport affecting metal and nutrient distributions and interfacial fluxes in permeable sediments. Geochim. Cosmochim. Acta 62, 613–631. doi: 10.1016/S0016-7037(97)00371-2

[ref34] Hug L. A. Castelle C. J. Wrighton K. C. Thomas B. C. Sharon I. Frischkorn K. R.. (2013). Community genomic analyses constrain the distribution of metabolic traits across the Chloroflexi phylum and indicate roles in sediment carbon cycling. Microbiome 1:22. doi: 10.1186/2049-2618-1-22, PMID: 24450983 PMC3971608

[ref35] Illumina. (2013). 16s metagenomic sequencing library preparation.

[ref36] Inagaki F. Nunoura T. Nakagawa S. Teske A. Lever M. Lauer A.. (2006). Biogeographical distribution and diversity of microbes in methane hydrate-bearing deep marine sediments on the Pacific Ocean margin. Proc. Natl. Acad. Sci. 103, 2815–2820. doi: 10.1073/pnas.0511033103, PMID: 16477011 PMC1413818

[ref37] Jansen S. Walpersdorf E. Werner U. Billerbeck M. Böttcher M. E. De Beer D. (2009). Functioning of intertidal flats inferred from temporal and spatial dynamics of O2, H2s and Ph in their surface sediment. Ocean Dyn. 59, 317–332. doi: 10.1007/s10236-009-0179-4

[ref38] Lever M. A. Alperin M. Engelen B. Inagaki F. Nakagawa S. Steinsbu B. O.. (2006). Trends in basalt and sediment Core contamination during Iodp expedition 301. Geomicrobiol J. 23, 517–530. doi: 10.1080/01490450600897245

[ref9002] Lueders T. Manefield M. Friedrich M. W. (2004). Enhanced sensitivity of DNA and rRNA-Based stable isotopeprobing byfractionation and quantitative analysis of isopycnic centrifugation gradients. Environmental Microbiology, 6, 73–78. doi: 10.1046/j.1462-2920.2003.00536.x 14686943

[ref39] Luef B. Frischkorn K. R. Wrighton K. C. Holman H.-Y. N. Birarda G. Thomas B. C.. (2015). Diverse uncultivated ultra-small bacterial cells in groundwater. Nat. Commun. 6:6372. doi: 10.1038/ncomms7372 25721682

[ref40] Luijendijk A. Hagenaars G. Ranasinghe R. Baart F. Donchyts G. Aarninkhof S. (2018). The state of the World’s beaches. Sci. Rep. 8:6641. doi: 10.1038/s41598-018-24630-6, PMID: 29703960 PMC5923213

[ref41] Lunau M. Lemke A. Walther K. Martens-Habbena W. Simon M. (2005). An improved method for counting Bacteria from sediments and turbid environments by epifluorescence microscopy. Environ. Microbiol. 7, 961–968. doi: 10.1111/j.1462-2920.2005.00767.x, PMID: 15946292

[ref42] Massmann G. Abarike G. Amoako K. Auer F. Badewien T. H. Berkenbrink C.. (2023). The Dynadeep observatory – a unique approach to study high-energy subterranean estuaries. Front. Mar. Sci. 10:1189281. doi: 10.3389/fmars.2023.1189281

[ref43] Moncada C. Arnosti C. Brüwer J. D. De Beer D. Amann R. Knittel K. (2024). Niche separation in bacterial communities and activities in Porewater, loosely attached, and firmly attached fractions in permeable surface sediments. ISME J. 18:wrae159. doi: 10.1093/ismejo/wrae159 39115410 PMC11368169

[ref44] Moore W. S. (1999). The subterranean estuary: a reaction zone of ground water and sea water. Mar. Chem. 65, 111–125. doi: 10.1016/S0304-4203(99)00014-6

[ref45] Moore W. S. (2010). The effect of submarine groundwater discharge on the ocean. Annu. Rev. Mar. Sci. 2, 59–88. doi: 10.1146/annurev-marine-120308-081019, PMID: 21141658

[ref46] Nelson W. C. Stegen J. C. (2015). The reduced genomes of Parcubacteria (Od1) contain signatures of a symbiotic lifestyle. Front. Microbiol. 6:713. doi: 10.3389/fmicb.2015.00713, PMID: 26257709 PMC4508563

[ref47] Parada A. E. Needham D. M. Fuhrman J. A. (2016). Every base matters: assessing small subunit Rrna primers for marine microbiomes with mock communities, time series and global field samples. Environ. Microbiol. 18, 1403–1414. doi: 10.1111/1462-2920.13023, PMID: 26271760

[ref48] Probandt D. Eickhorst T. Ellrott A. Amann R. Knittel K. (2017). Microbial life on a sand grain: from bulk sediment to single grains. ISME J. 12, 623–633. doi: 10.1038/ismej.2017.197 29192905 PMC5776476

[ref49] Reckhardt A. Beck M. Seidel M. Riedel T. Wehrmann A. Bartholomä A.. (2015). Carbon, nutrient and trace metal cycling in Sandy sediments: a comparison of high-energy beaches and Backbarrier tidal flats. Estuar. Coast. Shelf Sci. 159, 1–14. doi: 10.1016/j.ecss.2015.03.025

[ref50] Reckhardt A. Meyer R. Seibert S. L. Greskowiak J. Roberts M. Brick S.. (2024). Spatial and temporal dynamics of groundwater biogeochemistry in the deep subsurface of a high-Energy Beach. Mar. Chem. 267:104461. doi: 10.1016/j.marchem.2024.104461

[ref51] Rinke C. Schwientek P. Sczyrba A. Ivanova N. N. Anderson I. J. Cheng J.-F.. (2013). Insights into the phylogeny and coding potential of microbial dark matter. Nature 499, 431–437. doi: 10.1038/nature12352, PMID: 23851394

[ref52] Robinson C. Gibbes B. Carey H. Li L. (2007a). Salt-freshwater dynamics in a subterranean estuary over a spring-neap tidal cycle. J. Geophys. Res. Oceans 112. doi: 10.1029/2006JC003888

[ref53] Robinson C. Li L. Barry D. A. (2007b). Effect of tidal forcing on a subterranean estuary. Adv. Water Resour. 30, 851–865. doi: 10.1016/j.advwatres.2006.07.006

[ref54] Röper T. Kröger K. F. Meyer H. Sültenfuss J. Greskowiak J. Massmann G. (2012). Groundwater ages, recharge conditions and Hydrochemical evolution of a Barrier Island freshwater Lens (Spiekeroog, northern Germany). J. Hydrol. 454-455, 173–186. doi: 10.1016/j.jhydrol.2012.06.011

[ref55] RStudio-Team (2022). RStudio: Integrated development environment for R. Boston: Ma RStudio, Pbc.

[ref56] Ruiz-González C. Rodellas V. Garcia-Orellana J. (2021). The microbial dimension of submarine groundwater discharge: current challenges and future directions. FEMS Microbiol. Rev. 45:fuab010. doi: 10.1093/femsre/fuab010, PMID: 33538813 PMC8498565

[ref57] Ruiz-González C. Rodríguez-Pie L. Maister O. Rodellas V. Alorda-Keinglass A. Diego-Feliu M.. (2022). High spatial heterogeneity and low connectivity of bacterial communities along a Mediterranean subterranean estuary. Mol. Ecol. 31, 5745–5764. doi: 10.1111/mec.16695, PMID: 36112071 PMC9827943

[ref58] Rusch A. Forster S. Huettel M. (2001). Bacteria, diatoms and detritus in an intertidal sandflat subject to Advective transport across the water-sediment Interface. Biogeochemistry 55, 1–27. doi: 10.1023/A:1010687322291

[ref59] Sambrook J. (1989). Molecular cloning: a laboratory manual. Cold Spring Harbor, NY: Cold Spring Harbor Laboratory Press.

[ref60] Santos I. R. Eyre B. D. Huettel M. (2012). The driving forces of Porewater and groundwater flow in permeable coastal sediments: a review. Estuar. Coast. Shelf Sci. 98, 1–15. doi: 10.1016/j.ecss.2011.10.024

[ref61] Seidel M. Beck M. Riedel T. Waska H. Suryaputra I. G. N. A. Schnetger B.. (2014). Biogeochemistry of dissolved organic matter in an anoxic Intertidal Creek Bank. Geochim. Cosmochim. Acta 140, 418–434. doi: 10.1016/j.gca.2014.05.038

[ref62] Seidel M. Graue J. Engelen B. Köster J. Sass H. Rullkötter J. (2012). Advection and diffusion determine vertical distribution of microbial communities in intertidal sediments as revealed by combined biogeochemical and molecular biological analysis. Org. Geochem. 52, 114–129. doi: 10.1016/j.orggeochem.2012.08.015

[ref63] Simmons G. M. Jr. (1992). Importance of submarine groundwater discharge (Sgwd) and seawater cycling to material flux across sediment water interfaces in marine environments, vol. 84, 173–184.

[ref64] Tebbe D. A. Geihser S. Wemheuer B. Daniel R. Schäfer H. Engelen B. (2022). Seasonal and zonal succession of bacterial communities in North Sea salt marsh sediments. Microorganisms 10:859. doi: 10.3390/microorganisms10050859, PMID: 35630305 PMC9146408

[ref65] Waska H. Greskowiak J. Ahrens J. Beck M. Ahmerkamp S. Böning P.. (2019). Spatial and temporal patterns of pore water chemistry in the inter-tidal zone of a high Energy Beach. Front. Mar. Sci.:6:154. doi: 10.3389/fmars.2019.00154

[ref66] Waska H. Simon H. Ahmerkamp S. Greskowiak J. Ahrens J. Seibert S. L.. (2021). Molecular traits of dissolved organic matter in the subterranean estuary of a high-Energy Beach: indications of sources and sinks. Front. Mar. Sci. 8:607083. doi: 10.3389/fmars.2021.607083

[ref67] Wilms R. Köpke B. Sass H. Chang T. S. Cypionka H. Engelen B. (2006a). Deep biosphere-related Bacteria within the subsurface of tidal flat sediments. Environ. Microbiol. 8, 709–719. doi: 10.1111/j.1462-2920.2005.00949.x, PMID: 16584482

[ref68] Wilms R. Sass H. Köpke B. Köster J. Cypionka H. Engelen B. (2006b). Specific bacterial, archaeal, and eukaryotic communities in tidal-flat sediments along a vertical profile of several meters. Appl. Environ. Microbiol. 72, 2756–2764. doi: 10.1128/AEM.72.4.2756-2764.2006, PMID: 16597980 PMC1449071

[ref69] Zielinski O. Pieck D. Schulz J. Thölen C. Wollschläger J. Albinus M.. (2022). The Spiekeroog coastal observatory: a scientific infrastructure at the Land-Sea transition zone (southern North Sea). Front. Mar. Sci. 8:754905. doi: 10.3389/fmars.2021.754905

