# Supplementary material for: Interstitial microbial communities of coastal sediments are dominated by Nanoarchaeota
Source: Front Microbiol. 2025 Feb 18;16:1532193. doi: 10.3389/fmicb.2025.1532193 (PMC11876391; doi:10.3389/fmicb.2025.1532193)
Supplement: Supplementary file 1 [file Data_Sheet_1.PDF]

Supplementary Material

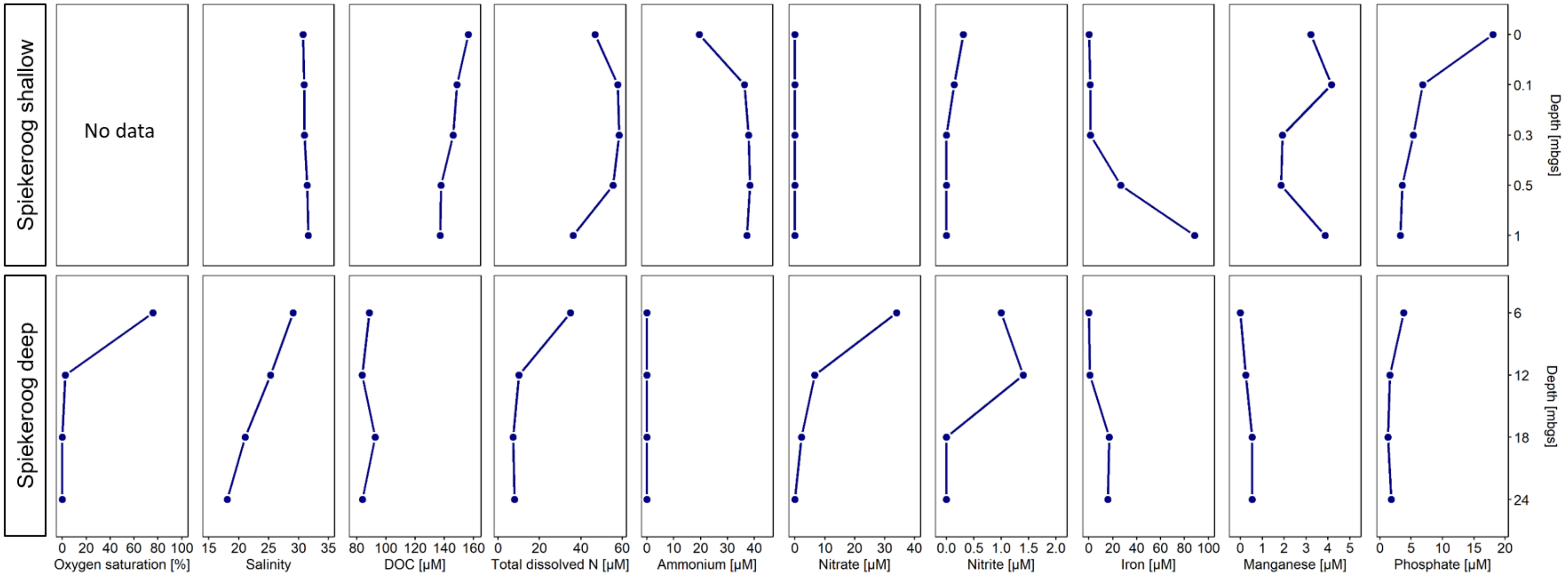

Supplementary Figure 1: Chemical composition of the interstitial porewaters of the shallow and deep Spiekeroog beach samples.

Supplementary Table 1: Chemical composition of the interstitial porewaters of the shallow and deep Spiekeroog beach samples.

Depth [mbgs]	O_2 [%]	Sal	DOC [µmol/l]	TDN [µmol/l]	NH_4 [µmol/l]	NO_3 [µmol/l]	NO_2 [µmol/l]	Fe [µmol/l]	Mn [µmol/l]	P [µmol/l]
0	NA	30.80	156.55	47.02	19.52	0.00	0.31	0.26	3.22	18.10
0.1	NA	30.97	148.73	58.07	36.47	0.00	0.14	1.15	4.17	6.87
0.3	NA	31.03	146.15	58.62	37.93	0.00	0.00	1.33	1.93	5.35
0.5	NA	31.50	137.83	55.71	38.45	0.00	0.00	26.79	1.87	3.58
1	NA	31.67	137.37	36.42	37.33	0.00	0.00	88.58	3.88	3.29
6	75.80	29.10	88.71	35.00	0.00	34.00	1.00	0.01	0.00	3.80
12	2.60	25.30	83.74	10.00	0.00	6.60	1.40	0.80	0.25	1.60
18	0.00	21.10	92.59	7.17	0.00	2.20	0.00	17.00	0.54	1.30
24	0.00	18.10	84.05	7.87	0.00	0.00	0.00	16.00	0.54	1.80

Supplementary Table 2: Cell numbers of microbial communities and porosity of shallow Spiekeroog beach samples, Janssand tidal-flat samples and deep Spiekeroog beach samples.

Spiekeroog shallow				Janssand tidal-flat		Spiekeroog deep			
Depth [mbgs]	Cells/cm^3 sediment	Cells/ml porewater	Porosity [%]	Cells/cm^3 sediment	Porosity [%]	Depth [mbgs]	Cells/cm^3 sediment	Cells/ml porewater	Porosity [%]
0	$1.2 \cdot 10^8$	$1.1 \cdot 10^6$	39	$3.3 \cdot 10^9$	30	6	$2.6 \cdot 10^6$	$5.4 \cdot 10^4$	24
0.1	$7.4 \cdot 10^7$	$1.0 \cdot 10^6$	11	$1.1 \cdot 10^9$	34	12	$1.0 \cdot 10^7$	$8.8 \cdot 10^5$	21
0.3	$1.2 \cdot 10^8$	$7.2 \cdot 10^5$	21	$1.9 \cdot 10^8$	36	18	$3.0 \cdot 10^6$	$8.4 \cdot 10^4$	18
0.5	$8.0 \cdot 10^7$	$6.5 \cdot 10^5$	19	$2.9 \cdot 10^8$	34	24	$5.0 \cdot 10^6$	$4.3 \cdot 10^4$	18
1	$8.1 \cdot 10^7$	$7.2 \cdot 10^5$	24	$5.8 \cdot 10^8$	53				

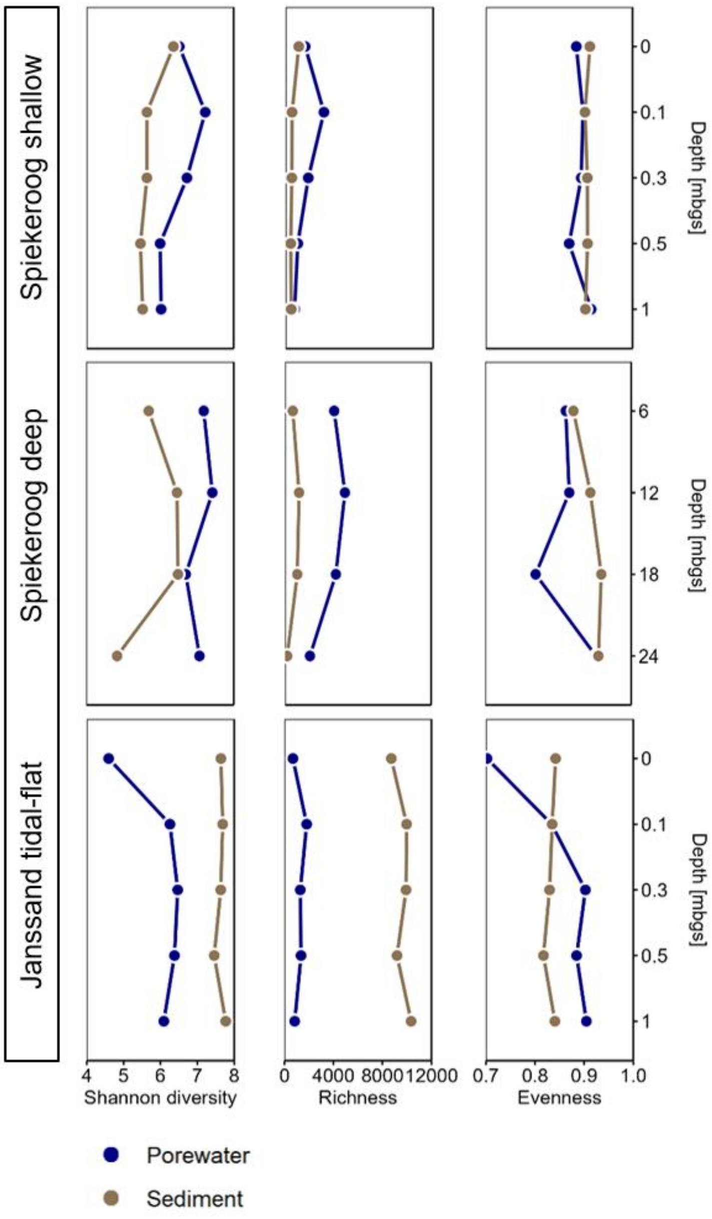

Supplementary Figure 2: Shannon diversity, richness and evenness of the shallow and deep beach samples and the Janssand tidal flat calculated on the basis of ASVs using Bray-Curtis dissimilarities.

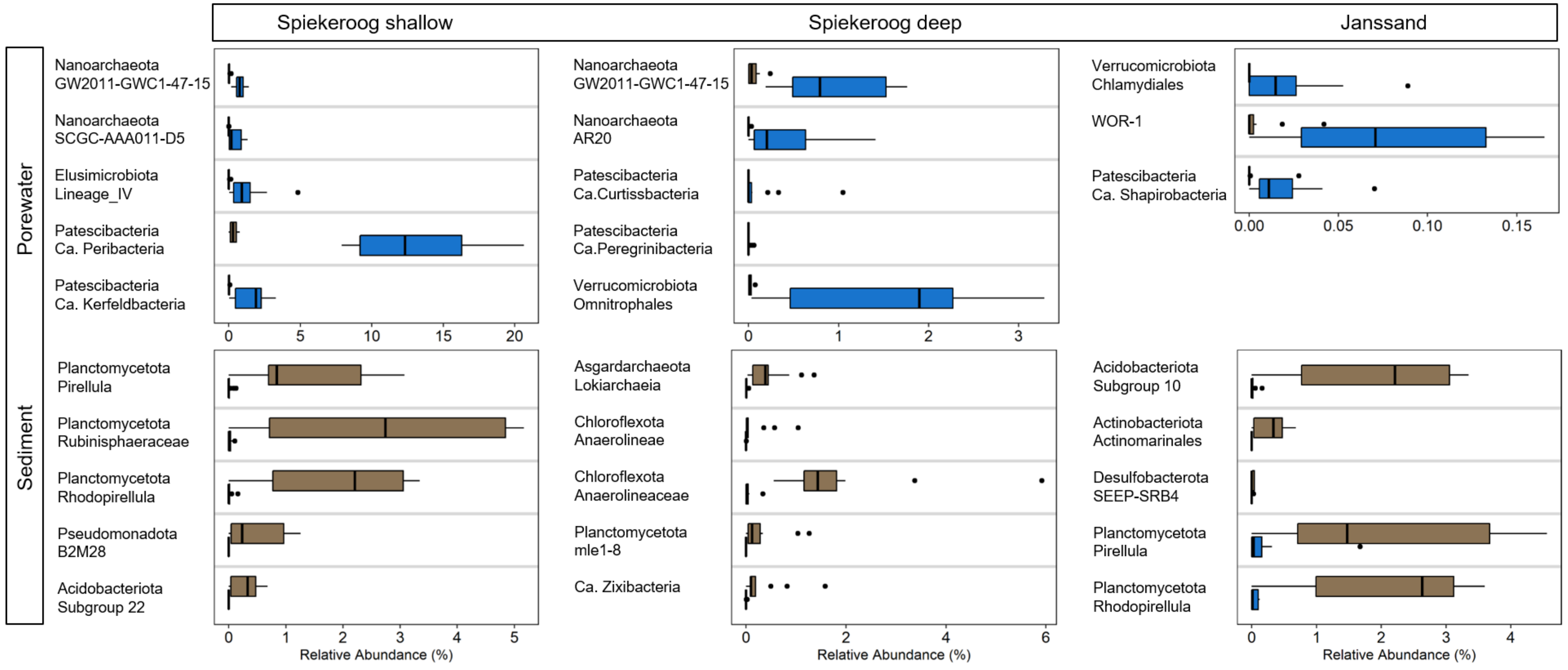

Supplementary Figure 3: Indicator species analysis of the shallow and deep beach and Janssand tidal flat communities. The boxplots show the five indicator species with the highest relative abundance and significant p-values (< 0.05) for porewater (blue) and sediment (brown). Only three taxa were strongly associated to the porewater of the Janssand tidal flat, but these taxa had very low abundances.

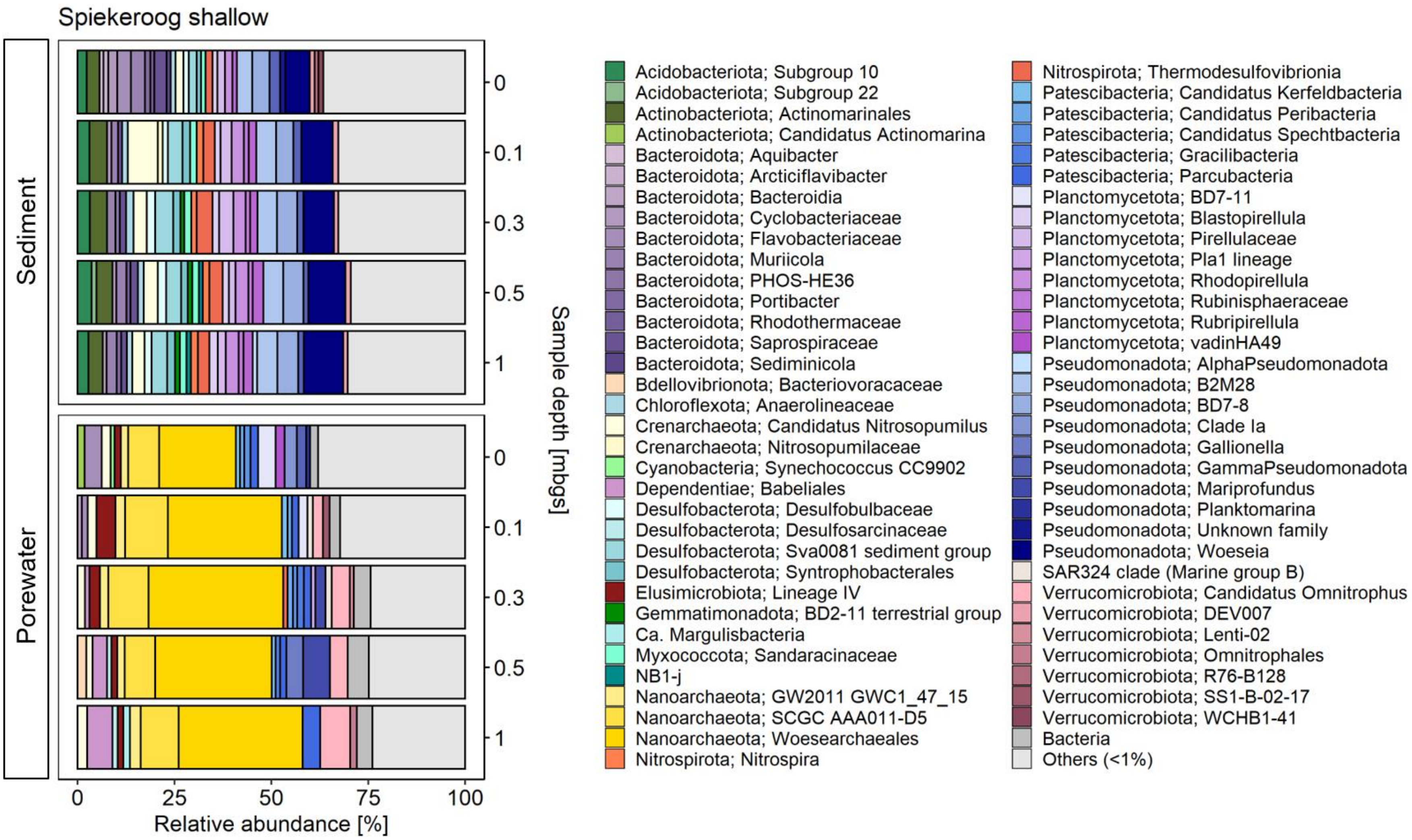

Supplementary Figure 4a: The barplots show the relative abundance of bacterial and archaeal taxa in the porewater and sediment of the different depths of the shallow beach samples. For the analyses the highest available taxonomic level was used. All taxa with ratios below 1% were summed up as “Others”.

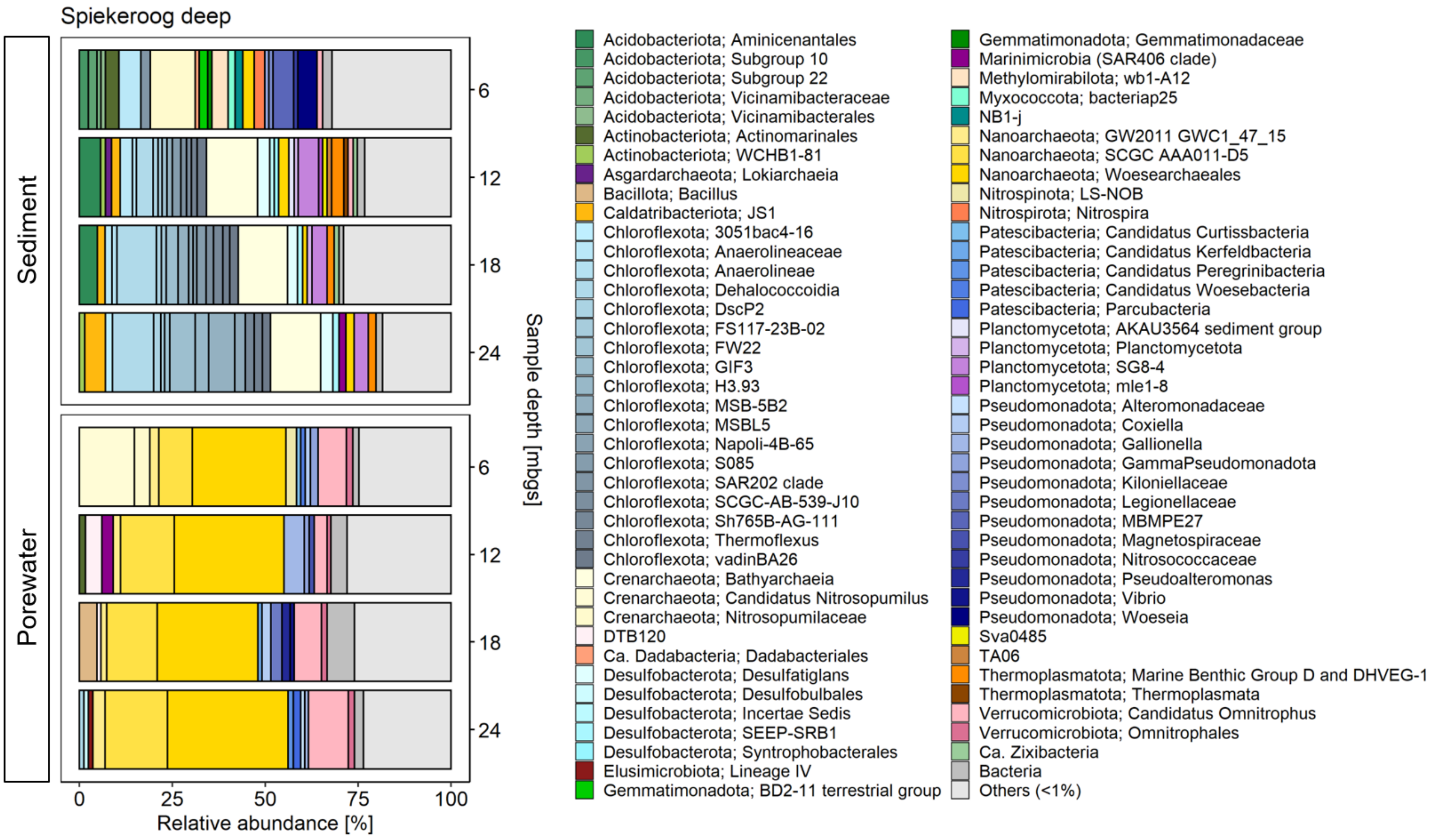

Supplementary Figure 4b: The barplots show the relative abundance of bacterial and archaeal taxa in the porewater and sediment of the different depths of the deep beach samples. For the analyses the highest available taxonomic level was used. All taxa with ratios below 1% were summed up as “Others”.

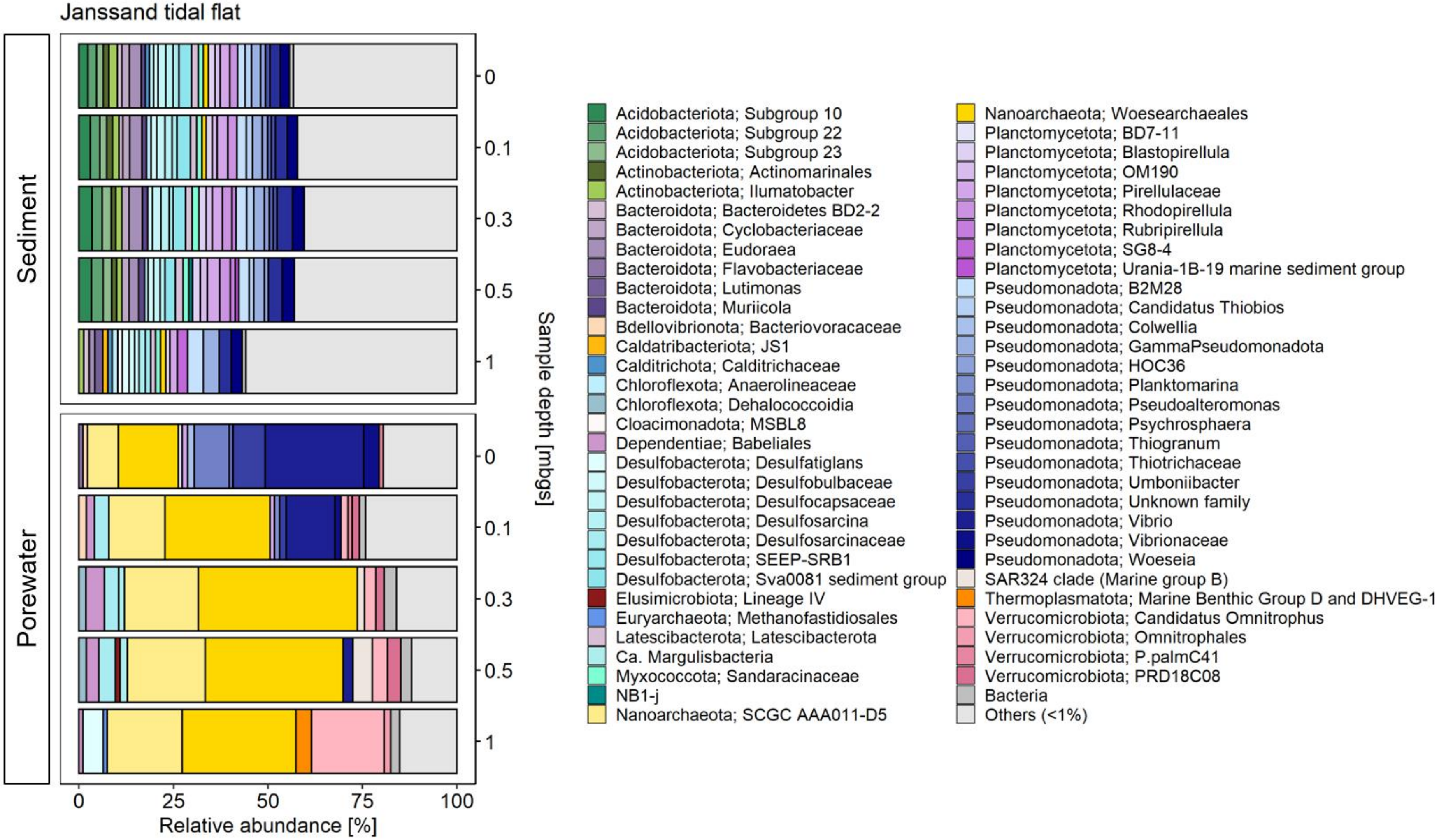

Supplementary Figure 4c: The barplots show the relative abundance of bacterial and archaeal taxa in the porewater and sediment of the different depths of the Janssand tidal-flat samples. For the analyses the highest available taxonomic level was used. All taxa with ratios below 1% were summed up as “Others”
